# FGF7–FGFR2 autocrine signaling increases growth and chemoresistance of fusion‐positive rhabdomyosarcomas

**DOI:** 10.1002/1878-0261.13145

**Published:** 2021-12-18

**Authors:** Christopher I. Milton, Joanna Selfe, Ewa Aladowicz, Stella Y. K. Man, Carolina Bernauer, Edoardo Missiaglia, Zoë S. Walters, Susanne A. Gatz, Anna Kelsey, Melanie Generali, Gary Box, Melanie Valenti, Alexis de Haven‐Brandon, David Galiwango, Angela Hayes, Matthew Clarke, Elisa Izquierdo, David Gonzalez De Castro, Florence I. Raynaud, Vladimir Kirkin, Janet M. Shipley

**Affiliations:** ^1^ Sarcoma Molecular Pathology Team Divisions of Molecular Pathology and Cancer Therapeutics The Institute of Cancer Research London UK; ^2^ Department of Paediatric Histopathology Manchester University NHS Foundation Trust Royal Manchester Children’s Hospital UK; ^3^ Cancer Pharmacology and Stress Response Team Division of Cancer Therapeutics The Institute of Cancer Research London UK; ^4^ Drug Metabolism and Pharmacokinetics Team Division of Cancer Therapeutics The Institute of Cancer Research London UK; ^5^ Glioma Team Division of Molecular Pathology The Institute of Cancer Research London UK; ^6^ Molecular Haematology Division of Molecular Pathology The Institute of Cancer Research London UK; ^7^ Present address: Signal Transduction and Molecular Pharmacology Team Cancer Research UK Cancer Therapeutics Unit The Institute of Cancer Research Sutton UK; ^8^ Present address: Center for Therapy Development and Good Manufacturing Practice Institute for Regenerative Medicine (IREM) University of Zurich Switzerland; ^9^ Present address: Department of Molecular Pathology Centre Hospitalier Universitaire Vaudois Lausanne Switzerland; ^10^ Present address: Translational Epigenomics Team Human Development and Health Faculty of Medicine Southampton General Hospital UK; ^11^ Present address: Institute of Cancer and Genomic Sciences University of Birmingham UK; ^12^ Present address: School of Medicine Dentistry and Biomedical sciences Queens University Belfast UK

**Keywords:** autocrine loop, FGF7, FGFR2, fibroblast growth factor receptor, NVP‐BGJ398, rhabdomyosarcoma

## Abstract

Rhabdomyosarcomas are aggressive pediatric soft‐tissue sarcomas and include high‐risk *PAX3–FOXO1* fusion‐gene‐positive cases. Fibroblast growth factor receptor 4 (FGFR4) is known to contribute to rhabdomyosarcoma progression; here, we sought to investigate the involvement and potential for therapeutic targeting of other FGFRs in this disease. Cell‐based screening of FGFR inhibitors with potential for clinical repurposing (NVP‐BGJ398, nintedanib, dovitinib, and ponatinib) revealed greater sensitivity of fusion‐gene‐positive versus fusion‐gene‐negative rhabdomyosarcoma cell lines and was shown to be correlated with high expression of FGFR2 and its specific ligand, FGF7. Furthermore, patient samples exhibit higher mRNA levels of *FGFR2* and *FGF7* in fusion‐gene‐positive versus fusion‐gene‐negative rhabdomyosarcomas. Sustained intracellular mitogen‐activated protein kinase (MAPK) activity and FGF7 secretion into culture media during serum starvation of *PAX3–FOXO1* rhabdomyosarcoma cells together with decreased cell viability after genetic silencing of *FGFR2* or *FGF7* was in keeping with a novel FGF7–FGFR2 autocrine loop. FGFR inhibition with NVP‐BGJ398 reduced viability and was synergistic with SN38, the active metabolite of irinotecan. *In vivo*, NVP‐BGJ398 abrogated xenograft growth and warrants further investigation in combination with irinotecan as a therapeutic strategy for fusion‐gene‐positive rhabdomyosarcomas.

## Graphical abstract

The PAX3‐FOXO1 fusion protein in high‐risk rhabdomyosarcomas increases *FGFR4, FGFR2,* and *FGF7* levels. We demonstrate FGF7, a specific ligand of FGFR2, forms an autocrine loop that signals through MAPkinase and contributes to cell proliferation/survival. This is disrupted by the Receptor Tyrosine Kinase inhibitor (RTKi) NVP‐BGJ398, which also affects FGFR4. NVP‐BGJ398 combined with irinotecan shows potential to treat fusion‐positive rhabdomyosarcoma.

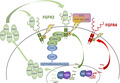

**Abbreviations**
AKT: AKT serine threonine kinase
ARMS: alveolar rhabdomyosarcoma
cDNA: complementary deoxyribonucleic acid
CHEK1: checkpoint kinase 1
COG/IRSG: Children’s Oncology Group/Intergroup Rhabdomyosarcoma Study Group
DNA: deoxyribonucleic acid
ELISA: enzyme‐linked immunosorbent assay
ERK: extracellular signal‐regulated kinase
ERMS: embryonal rhabdomyosarcoma
FANCD2: fanconi anemia complementation group D2
FBS: fetal bovine serum
FGF: fibroblast growth factor
FGFR: fibroblast growth factor receptor
FN‐RMS: fusion‐gene‐negative rhabdomyosarcoma
FOXO1: forkhead box O1
FP‐RMS: fusion‐gene‐positive rhabdomyosarcoma
FRS2α: fibroblast growth factor receptor substrate 2α
GAPDH: glyceraldehyde‐3‐phosphate dehydrogenase
GI_50_
: growth inhibition at 50%
H2A.X: H2A histone family member X
IHC: immuohistochemistry
ITCC/CIT: Innovative Therapies for Children with Cancer/Carte d’Identite’ des Tumeurs
MAPK: mitogen‐activated protein kinase
mRNA: messenger ribonucleic acid
mTOR: mammalian target of rapamycin
MTS: methyl tetrazolium salt
NTC: nontargeting control
P3F: PAX3‐FOXO1
P7F: PAX7‐FOXO1
PARP: poly (ADP‐Ribose) polymerase
PAX3: paired box 3
PDGF: platelet derived growth factor
PDX: patient derived xenograft
PI3K: phosphatidylinositol‐3 kinase
q.d.: Quaque die
qRT‐PCR: quantitative reverse‐transcription polymerase chain reaction
RAS: rat sarcoma viral oncogene
RMS: rhabdomyosarcoma
RTKs: receptor tyrosine kinases
siRNA: small interfering ribonucleic acid
STAT3: signal transducer and activator of transcription 3
TMA: tissue microarray
UTC: untransfected control

## 1 Introduction

The fibroblast growth factor receptor family comprises four functional members (FGFRs 1‐4) that bind with varying affinities to 22 different fibroblast growth factor (FGF) ligands to activate downstream signaling pathways controlling cell proliferation, differentiation, vasculature, survival, and migration [1, 2]. In this way, FGF/R signaling has been shown to control cardiac, lung and muscle development, wound healing, and embryonic patterning [3, 4, 5, 6, 7, 8, 9]. In cancers, FGF/R signaling is frequently deregulated leading to tumor cell growth, survival, and metastasis [10, 11, 12, 13]. Rhabdomyosarcomas (RMS) are the most common pediatric soft‐tissue sarcoma with features resembling developing skeletal muscle and previous investigations indicate that FGF/R signaling plays a role in this disease.

*FGFR1* amplification and elevated expression was reported in 3% of fusion‐negative rhabdomyosarcoma (FN‐RMS) patient samples and cell lines and in another study *FGFR1* overexpression in patients was attributed to hypomethylation of CpG islands upstream of exon 1 [14, 15]. In fusion‐positive rhabdomyosarcoma (FP‐RMS), high levels of FGFR4 expression are transcriptionally driven by the protein product of the recurrent fusion gene *PAX3*‐*FOXO1* [16, 17]. Intriguingly, while evidence demonstrates that wild‐type FGFR4 controls proliferation, migration, and survival of FP‐RMS cells, constitutively active mutations in FGFR4 control the proliferation of subsets of FN‐RMS cells [17, 18, 19, 20, 21]. Also, *FGFR2* was among the highest expressed genes in a patient derived xenograft (PDX) model from a FP‐RMS patient heavily pretreated with chemotherapy [22]. Despite this evidence for FGFRs, little is known about the expression or activity of FGF ligands in RMS.

While targeting FGFR4 has been proposed as a rational therapeutic strategy in RMS, it is not clear which patients would benefit most from this or whether targeting other FGFR members would be beneficial. We therefore sought further evidence for the involvement of FGFs and FGFRs in RMS biology and the potential of four clinically relevant FGFR inhibitors [23, 24, 25, 26] alone and in combination with standard chemotherapeutic agents for treating RMS.

## 2 Materials and methods

### 2.1 Cell culture and reagents

Human RMS cell lines used included RMS01, RH4 (CVCL_5916), SCMC, RH41 (CVCL_2176), RH30 (CVCL_0041), RMS‐YM (CVCL_A792), RMS559 (CVCL_S640), RD (CVCL_1649), JR1 (CVCL_J063), TE617.T (CVCL_1755), and RUCH3 (CVCL_C541), which were from Fred G. Barr (Center for Cancer Research, NCI, Bethesda) and Corinne Linardic (Duke University School of Medicine, Durham, NC) and have been described previously [21, 27] with the addition of SMS‐CTR (CT10; CVCL_A770) (from Timothy, J. Triche, Keck School of Medicine, Los Angeles, CA) and TE381.T (CVCL_1751) cells (from Marcel Kool, DKFZ, Heidelberg, Germany). Authentication was confirmed by Short Tandem Repeat (STR) analysis using the GenePrint 10 kit (Promega, Madison, WI, USA). Only RH41 and RMS‐YM were cultured in RPMI, the rest in DMEM, supplemented with 10% fetal bovine serum (FBS), 2 mm L‐glutamine, and 1% penicillin/streptomycin (Thermo Scientific, Waltham, MA, USA). Cells were maintained at 37 °C in 5% CO_2_ and were routinely tested for infection/mycoplasma using the HEK‐Blue 2 cell system (Invivogen Cat#rep‐pt1, San Diego, CA, USA). The following compounds were from Selleck Chemicals (Houston, TX, USA) PD‐173074 (Cat#S1264), ponatinib (Cat#S1490), NVP‐BGJ398 (Cat#S2183), dovitinib (Cat#S1018), nintedanib (Cat#S1010), SN38 (Cat#S4908), and vincristine sulfate (Cat#S1241) and were dissolved to 20 mm stock in DMSO (Sigma‐Aldrich, Cat#D2650, Gillingham, Dorset, UK). cis‐Diamineplatinum(II) dichloride (Sigma‐Aldrich, Cat#479306) was dissolved to 2 mm in sterile PBS. Recombinant human FGF7 (Peprotech Cat#100‐19, Cranbury, NJ, USA) and FGF19 (Peprotech Cat#100‐32) were reconstituted to 0.1 mg·mL^−1^ in PBS with 0.1% BSA as a carrier protein.

### 2.2 Methyl Tetrazolium Salt (MTS) assays

Cells were seeded at 2–5 × 10^3^ per well in clear 96‐well plates (Corning Cat#3596, Corning, NY, USA) and grown for 24 h before adding vehicle (0.1% DMSO final) or serial dilutions of compounds in media. Cells were cultured for 72 h for drug screening or for 144 h for combination assays before addition of CellTiter96 Aqueous One according to the manufacturers’ instructions (Promega Cat#G3580). GI_50_s were calculated in graph pad prism 7 (Graph Pad Inc., San Diego, CA, USA) using nonlinear regression analysis with a variable slope. Combination effects were assessed using the Bliss independence model, which calculates the difference between observed and expected fractional inhibition of drug combinations. Differences > 0 ≤ 1 are synergistic. All assays were conducted in biological triplicate.

### 2.3 Clonogenic assay

Cells were seeded at 5–10 × 10^3^ cells per well of a 24‐well plate (Thermo Scientific Cat#142475) and grown for 2 days before exposure to drugs for 5 days. Media was replaced and cells grown for a further 7 days before fixation and crystal violet staining as previously described [28]. Assays were run in biological quadruplicate.

### 2.4 Patient gene expression analysis

Our gene expression profiling data for 101 RMS patients (ArrayExpress database accession ID E‐TABM‐1202) and 36 skeletal muscle samples (from various public datasets) using the Affymetrix U133 Plus2.0 platform were normalized as previously described [15, 29]. Gene expression profiling data for a further 134 RMS patients (GEO accession GSE92689) and 34 skeletal muscle samples (from various public datasets) using the Affymetrix U133A platform were also analyzed. The median value for representative probe sets was compared between skeletal muscle and RMS patient subgroups to determine over or under expression.

### 2.5 Sequencing analysis

The cell lines RD and RMS559 were sequenced using a capture‐customized pediatric panel of 78 genes (Secap EZ, Nimblegen, Pleasanton, CA, USA). Libraries were prepared from 200 ng of DNA using the KAPA Hyper kit (Roche Cat#07962363001, Burgess Hill, West Sussex, UK) and SeqCap EZ Prime choice adapters (Roche). DNA was amplified, multiplexed, and hybridized using 1 µg of total precapture library DNA after which amplification and sequencing were conducted on a MiSeq (Illumina, San Diego, CA, USA) with 75 bp paired‐end reads following the manufacturer’s instructions. Analysis was completed using miseq reporter software (Illumina) with alignment against the human reference sequence GRCh37/Hg19. Median read depth was 447×.

### 2.6 siRNA transfection

Cells were grown overnight at 2 × 10^3^ cells per well in a 96‐well plate or 1.25 × 10^5^ cells per well in a 6‐well plate (Corning Cat#353046) without penicillin/streptomycin. Three independent siRNAs targeting *FGFR2* (Qiagen Cat#SI02623047, SI04380649, and SI04948909, Hilden, Germany) or *FGF7* (Qiagen Cat#SI03064663, SI03074925, and SI03110163) were transfected alongside an AllStars Negative (Qiagen Cat#1027281) and Hs cell death control (Qiagen Cat#1027299) at a final concentration of 25 nm using RNAimax (Thermo Scientific Cat#13778075) according to the manufacturer’s instructions. Cell viability was assessed after 144 h or were harvested after 72 h in 1xRIPA lysis buffer (Cell Signaling Technologies Cat# 9806S, Danvers, MA, USA) or Trizol (Thermo Scientific Cat#15596018) for protein and qRT‐PCR assessment, respectively. Assays were in biological triplicate.

### 2.7 Quantitative real‐time PCR

Trizol extracted total RNA was subjected to cDNA synthesis using the High Capacity Reverse Transcription Kit (Applied Biosystems Cat#4368814, Waltham, MA, USA). Predesigned FAM‐labeled Taqman assays (all Thermo Scientific) to *FGFR1* (Hs00241111_m1), *FGFR2* (Hs01552926_m1), *FGFR3* (Hs00179829_m1), *FGFR4* (Hs01106908_m1), or *FGF7* (Hs00940253_m1) were run in biological triplicate on a Viia7 Real‐time PCR system (Thermo Scientific), according to the manufacturer’s instructions with a VIC‐TAMRA‐labeled *β‐actin* assay (Thermo Scientific Cat#4310881E) as an internal control. Quantities of RNA per well were interpolated from a standard curve, normalized to the internal control, and then normalized to control samples as indicated.

### 2.8 Immunoprecipitation

Cells exposed to NVP‐BGJ398 for 3 h and either 25 ng·mL^−1^ FGF7 or 150 ng·mL^−1^ FGF19 for 20 min were harvested in 1xRIPA containing Complete mini protease inhibitor (Roche Cat#4693159001) and Phosphatase Inhibitor cocktails (Sigma Aldrich Cat#P0044 and P5726). 1.2 mg of protein lysate was precleared with 1.5 mg Protein A Dynabeads (Thermo Scientific Cat#10001D) on a rotator for 2 h at 4 °C before pulldown with 4 μg of either FGFR2 (Santa Cruz Biotechnologies, Cat#sc‐122, RRID:AB_631509), FGFR4 (Santa Cruz Biotechnologies, Cat#sc‐124, RRID:AB_631512), or normal rabbit IgG (Cell Signaling Technologies, Cat#2729, RRID:AB_1031062) as described previously [30].

### 2.9 Immunoblotting

Cells in 10% FBS were exposed to the indicated concentrations of drug for 3 h. Cell starvation (0% FBS) was achieved through removal of media, washing twice in PBS and then addition of basal media supplemented with L‐Glut and Pen/Strep (as above) but no FBS. Cells were left for 16 h prior to drug exposure for 3 h, the last 20 min of which included addition of FGF7 where indicated. Cells were harvested in 1× RIPA lysis buffer (containing protease and phosphatase inhibitors) before 30 μg of protein was resolved on 4–12% Bis‐Tris polyacrylamide gels (Thermo Scientific Cat#NP0323BOX), transferred onto nitrocellulose membranes (Thermo Scientific Cat#LC2000), and blocked in 5% milk then probed overnight at 4 °C with the antibodies listed in Table S1. Phosphorylated proteins were removed using Restore PLUS Western Blot stripping buffer (Thermo Scientific Cat#46430) prior to washing in 5% milk and subsequent re‐probing for totals. Proteins were detected using ECL Prime reagent (GE Healthcare Cat#RPN2232, Chicago, IL, USA) and quantified using Image Lab Touch software (Bio‐Rad, Hercules, CA, USA). Subcellular fractionation was achieved using the Subcellular protein fractionation kit for cultured cells (Thermo Scientific Cat#78840) according to the manufacturer’s instructions.

### 2.10 Electrochemiluminescent (MSD) assay

Electrochemiluminescent immunoassay plates were run with three biological replicates containing either 10 μg of *in vitro* or 80 μg of *in vivo* lysate to assess FRS2α Y196 phosphorylation (Meso Scale Discovery Cat#K150KJD‐1, Rockville, MD, USA) or with 40 μg of *in vivo* lysate to assess ERK1/2 T202/Y204 phosphorylation (Meso Scale Discovery Cat#K15107D‐1). Both plates were run according to the manufacturer’s instructions, except for an overnight incubation with primary antibody at 4 °C.

### 2.11 FGF7 enzyme‐linked immunosorbent assay (ELISA)

Cell culture media was collected on three separate occasions with 100 µL of each subjected to a FGF7 ELISA assay (R&D Systems Cat#DKG00, Minneapolis, MN, USA), which was performed according to the manufacturer’s instructions. Absorbance was read at 450 nm with a correction at 540 nm on a FLUORstar Optima plate reader (BMG‐Labtech, Aylesbury, Buckinghamshire, UK), and samples were quantified by interpolation from a standard curve of recombinant human FGF7 (kit supplied).

### 2.12 *In vivo* experiments

All animal studies and breeding were approved by the institutional Animal Welfare Ethical Review Body (AWERB) and carried out in accordance with UK Home Office Regulations under the Animals (Scientific Procedures) Act 1986 and national guidelines [31]. Mice were housed in individually ventilated cages (IVCs) within a specific‐pathogen‐free (SPF) facility and were monitored daily for welfare reasons with all experiments carried out under license PPL70/7635 (70). RMS01 and RH41 cells were injected into female 6‐ to 8‐week‐old NOD.CB17‐*Prkdc^scid^*/NcrCrl (NOD‐SCID) mice (Charles Rivers) at 2.5 × 10^6^ cells per site. Once tumors reached ~ 100 mm^3^, mice were randomly assigned to groups (*n* = 3 for PD/PK; *n* = 10 for efficacy) and received either vehicle for NVP‐BGJ398 (0.2 m sodium acetate in acetic acid [pH6.8] dissolved 1 : 1 in PEG300) or NVP‐BGJ398 by oral gavage. For the pharmacodynamic (PD) study, mice bearing RMS01 xenografts were dosed with either vehicle, 15 mg·kg^−1^ or 30 mg·kg^−1^ NVP‐BGJ398 prior to tumor harvesting 3 or 24 h later. Tumors were snap frozen and homogenized in 1× RIPA buffer (containing protease and phosphatase inhibitors) using a Precellys 24 (Bertin Instruments, Montigny‐le‐Bretonneux, France).

For the efficacy study, mice bearing RMS01 or RH41 xenografts were dosed orally with 30 or 25 mg·kg^−1^ NVP‐BGJ398 quaque die (q.d.) for 18 or 20 days, respectively. Mouse body weight and tumor volumes were measured 3 times weekly with the latter calculated from caliper measurements using the following formula: *V* = 4/3*π* [(*d*_1_ + *d*_2_)/4]^3^. Pharmacokinetic analysis was conducted on tumors and plasma 3 h after the last dose with compounds being quantified using liquid chromatography tandem mass spectrometry (LC‐MS/MS) with multiple reaction monitoring (MRM) and external calibration as in [32].

### 2.13 Immunohistochemistry

Tumor xenografts were fixed in 10% neutral buffered formalin (Sigma‐Aldrich, Cat#HT501128) for 24 h before dehydration, paraffin‐wax embedding, and being sectioned (3 μm) onto Superfrost Plus slides (Fisher Scientific Cat#J1800AMNT). RMS tissue microarray (TMA) slides containing patient material were as previously described [33]. Written consent was obtained for patient samples, which were handled according to Local Research Ethics Committee protocol 1836 and Multi‐regional Research Ethics Committee protocol 98/4/023. After dewaxing, rehydration, and washing, slides underwent heat‐induced antigen retrieval in 10 mm sodium citrate (pH 6.0) at 95 °C for 50 min prior to blocking for 1 h and then incubation with 1 : 500 anti‐FGFR2 (Santa Cruz Biotechnology, Cat#sc‐122, RRID:AB_631509) or 1 : 150 anti‐Ki67 (clone MIB‐1 Agilent Cat#M724029‐2, RRID:AB_2687528, Santa Clara, CA, USA) for 1 h at RT. Slides were washed, incubated in secondary antibody for 30 min, and then visualized by Dako REAL™ DAB+ Chromagen (DAKO Cat#K500711‐2, Glostrup, Denmark) for 3 min prior to counterstaining with Mayer's hematoxylin (DAKO Cat#S330930‐2). Slides were scanned on an Ariol microscope system (Leica, Wetzlar, Germany) and assessed blind by a practicing pathologist and an independent researcher. The percentage of cells positive for FGFR2 was scored as follows: < 10% = 0, 10–25% = 1, > 25% < 50% = 2, and ≥ 50% = 3, whereas Ki67 staining was expressed as the percentage of cells with positive immunostaining.

### 2.14 Statistics

All *in vitro* experiments were conducted in at least biological triplicate except for western blots, which were performed twice. The mean and standard deviation is shown unless stated otherwise and specific tests are as indicated in figure legends.

## 3 Results

### 3.1 FP‐RMS cells are sensitive to FGFR inhibitors

**Fig. 1:**
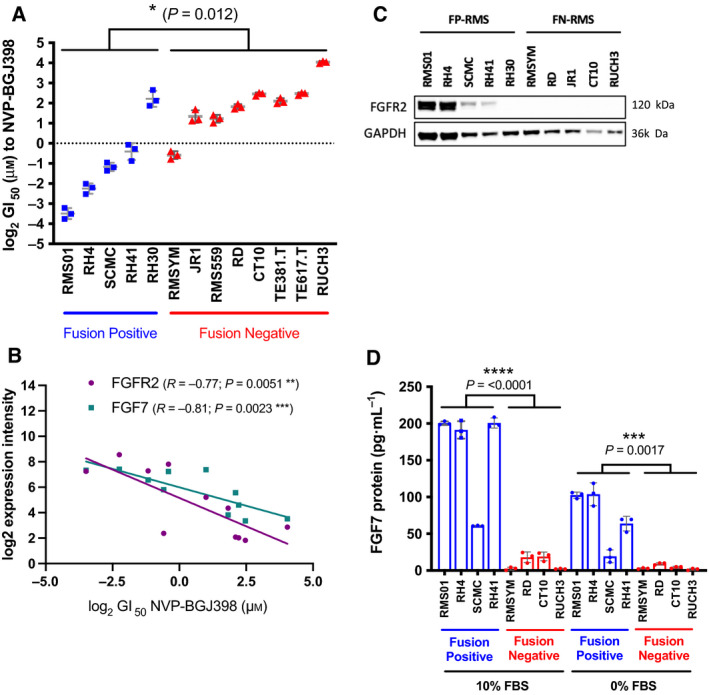
Sensitivity of PAX3‐FOXO1 fusion‐positive cells to NVP‐BGJ398 correlates with FGFR2 and FGF7 expression. (A) 2D Rhabdomyosarcoma (RMS) cell line Growth Inhibition at 50% (GI_50_) values as measured by Methyl Tetrazolium Salt (MTS) after 72 h exposure to NVP‐BGJ398. (B) Scatter plot of *FGFR2* (plum) and *FGF7* (teal) mRNA expression against the log2 GI_50_ to NVP‐BGJ398 in RMS cell lines. Each point represents a separate cell line. Spearman r correlation and associated *P* values with line of best fit (solid) are shown. (C) Representative western blot of FGFR2 protein in fusion‐positive (FP) and fusion‐negative (FN) RMS cells cultured in 10% Fetal Bovine Serum (FBS). (D) RMS cells were incubated with 10% or 0% FBS (serum starved) for 24 h before culture media was collected and subjected to FGF7 enzyme‐linked immunosorbent assay (ELISA). Results are representative of three independent experiments with error bars representing standard deviation, except for (C), which was repeated twice. Significance of differences were assessed using unpaired *t*‐tests.*P < 0.05, **P < 0.01, ***P < 0.005, ****P < 0.001.

Initial data using the tool compound PD‐173074 showed that FP‐RMS cells were significantly more sensitive to FGFR inhibition than FN‐RMS cells except for RH30 (Fig. S1A). Similarly, upon expanding our screen to 13 RMS lines (5 *PAX3‐FOXO1* FP and 8 FN) against four clinically relevant FGFR inhibitors, FP‐RMS cells were markedly more sensitive than FN‐RMS cells (ponatinib *P* = 0.014, NVP‐BGJ398 *P* = 0.012, dovitinib *P* = 0.004, and nintedanib *P* = 0.0008) (Fig. S1B). The most potent drug was ponatinib, but recent evidence has raised concerns over cardiovascular, pulmonary, and metabolic toxicities observed with its clinical use [34, 35, 36]. We therefore selected the next most potent compound in our screen NVP‐BGJ398, especially as available data indicated that this compound was the most FGFR selective inhibitor in biochemical assays (Table S2) [37, 38, 39, 40]. Most FP‐RMS cells were more sensitive to NVP‐BGJ398 than FN‐RMS cells except for the FP line RH30 and FN line RMS‐YM (Fig. 1A and Table S3).

### 3.2 Sensitivity to NVP‐BGJ398 correlates with FGFR2 and FGF7 expression

We assessed correlations between mRNA expression, mutation, or DNA copy number status in RMS cell lines with GI_50_s to NVP‐BGJ398. cDNA microarray expression of *FGFR2* and its ligand *FGF7* showed a strong inverse correlation with GI_50_ values to NVP‐BGJ398 in RMS cells (Fig. 1B). These mRNA expression levels were validated in our cell lines using quantitative real‐time PCR (qRT‐PCR) with strong concordance between the two assays (Fig. S2A,B). Furthermore, we identified a strong positive correlation between FGFR2 and FGF7 mRNA expression in RMS cell lines with higher expression of both ligand and receptor in FP‐RMS cells compared to FN‐RMS cells (Fig. S2C). The only exception to this were RH30 cells, which are fusion positive but have low expression of both genes and are less sensitive to FGFR inhibitors, despite high expression of FGFR4 (Fig. S2F).

We observed no significant correlation between NVP‐BGJ398 response and *FGFR1* or *FGFR3* mRNA expression across our cell panel (Fig. S2D,E) although *FGFR1* amplified RMS‐YM cells [15] exhibited high *FGFR1* mRNA expression and were the most sensitive FN‐RMS cell line tested (Fig. S2C). We also identified a strong correlation between *FGFR4* expression and sensitivity to NVP‐BGJ398 (Fig. S2E). Targeted re‐sequencing of RMS lines confirmed the V550L *FGFR4* mutation in RMS559 cells as previously described [21] but also identified an activating E69K mutation in the oncogenic phosphatase *PTPN11*. This mutation has been shown to stimulate Rat Sarcoma Viral Oncogene (RAS) and Extracellular Signal‐Regulated Kinases1/2 (ERK) signaling, causing resistance to RTK targeted therapies [41, 42] and alongside *NRAS* (Q61H) and *HRAS* (Q61K) mutations in the FN‐RMS cell lines RD and SMS‐CTR (CT10), respectively, is a likely cause of decreased sensitivity to NVP‐BGJ398.

In concordance with mRNA expression, we identified elevated FGFR2 and FGF7 protein in FP‐RMS cells and media, respectively, compared to FN‐RMS (Fig. 1C,D). Intriguingly, FGF7 was present in the media of FP‐RMS cells despite complete growth factor starvation (0% FBS) for 24 h, indicating that FGF7 is likely to be secreted from these cells. FGF7 preferentially binds to FGFR2 above other FGFR family members [1], and we hypothesized that as both are overexpressed in FP‐RMS cells this might lead to an autocrine loop, contributing to drug sensitivity.

### 3.3 *FGFR2* and *FGF7* expression is high in fusion‐positive RMS patients

**Fig. 2:**
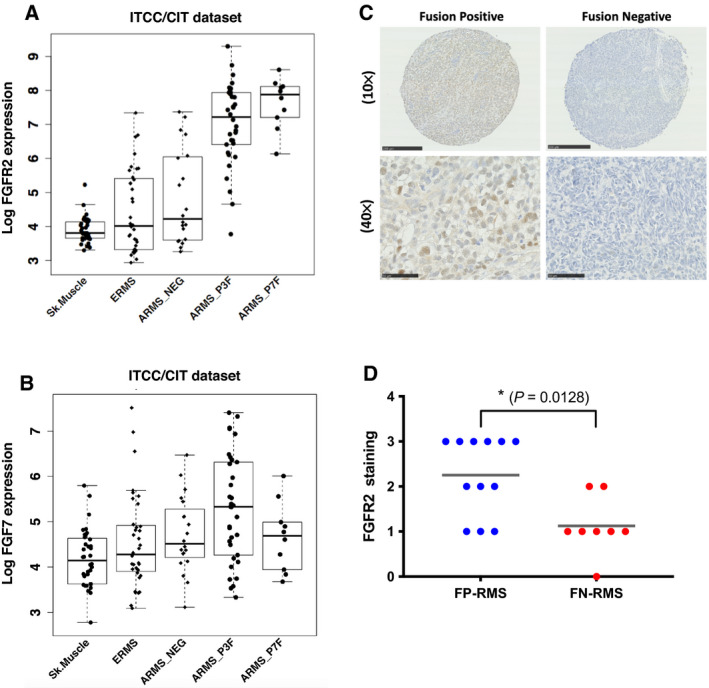
FGF7 mRNA and FGFR2 mRNA and protein is highly expressed in FP‐RMS patients. *FGFR2* (A) and *FGF7* (B) mRNA expression in Rhabdomyosarcoma (RMS) patients from the Innovative Therapies for Children with Cancer/Carte d’Identite’ des Tumeurs (ITCC/CIT) cohort. Sk. Muscle = skeletal muscle, ERMS = embryonal RMS, ARMS = alveolar RMS, NEG = negative, P3F = PAX3‐FOXO1 and P7F = PAX7‐FOXO1. Wilcoxon rank sum test, for P3F *FGFR2* is *P* < 0.001 and *FGF7* is *P* < 0.01 compared to fusion‐negative (FN‐RMS) samples. (*n* = 101 tumor and 36 normal samples) (C) Representative images of FGFR2 protein expression in a subset of RMS patients on a Tissue Microarray (TMA) by Immunohistochemistry (IHC). Magnification is 10× and 40× with scale bars representing 250 and 50 µm respectively. (*n* = 12 fusion‐positive and 8 fusion‐negative samples). (D) Quantification of the percentage of FGFR2 positive cells from the TMA IHC was as follows < 10% = 0, 10–25% = 1, > 25% < 50% = 2, ≥ 50% = 3 with scores compared between fusion‐positive (FP‐RMS; blue) and fusion‐negative (FN‐RMS; red) patients using a Mann‐Whitney *U* test. *P < 0.05; (*n* = 12 FP‐RMS and 8 FN‐RMS samples).

To determine the clinical relevance of FGFR2 and FGF7 in RMS patient tumors, we analyzed gene expression data generated in the ITCC/CIT (Innovative Therapies for Children with Cancer/Carte d’Identite’ des Tumeurs) cohort [29]. Both *FGFR2* and *FGF7* mRNA expressions were significantly greater in FP‐RMS tumors, compared with FN‐RMS tumors and normal skeletal muscle (Fig. 2A,B) (Wilcoxon rank‐sum test for FGFR2, *P* < 0.001; for FGF7, *P* < 0.01). These results were confirmed in a second patient cohort from the COG/IRSG (Children’s Oncology Group/Intergroup Rhabdomyosarcoma Study Group) [43] and an independent publicly available RNA‐Seq dataset [17] (Fig. S3). Patient FGFR2 protein expression was assessed by immunohistochemistry (IHC) on samples on a tissue microarray with moderate staining overall but significantly higher levels in FP‐RMS compared to FN‐RMS patients (Mann–Whitney *U* test; *P* = 0.0128) (Fig. 2C,D). Interestingly, nuclear FGFR2 was observed in FP‐RMS patient samples and cell lines alike (Fig. S4) with subcellular fractionation of the latter revealing full length (120 kDa) and shorter forms (60 kDa) in both membrane and soluble nuclear fractions (Fig. S4B). Both sets of bands were markedly reduced upon FGFR2 knockdown with siRNA confirming their specificity as FGFR2. The function of these shorter and nuclear forms of FGFR2 is currently not clear and being investigated further. Overall, these data demonstrate that overexpression of FGF7 and FGFR2 in FP‐RMS patient tumors and cell lines is indicative of an autocrine loop, which, *in vitro,* correlates with sensitivity to NVP‐BGJ398.

### 3.4 FGF7‐stimulated signaling is abrogated by NVP‐BGJ398 in FP‐RMS cells

**Fig. 3:**
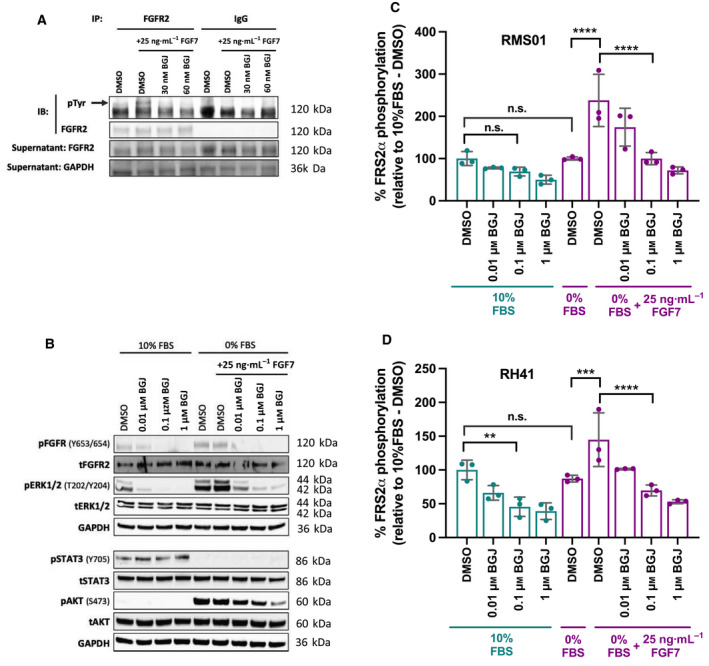
Molecular effects of NVP‐BGJ398 in RMS cell lines *in vitro*. (A) Representative blot assessing tyrosine phosphorylation on FGFR2 (arrow indicates 120 kDa). RMS01 cells were exposed to 3 h vehicle or drug before 20 min of 25 ng·mL^−1^ FGF7 and subsequent immunoprecipitation (IP) with a FGFR2 or IgG (Isotype control) antibody. The supernatant of remaining proteins after IP were retained and run as a control to demonstrate depletion in FGFR2 IP lanes compared to IgG control with GAPDH demonstrating equal loading. (B) Representative blot assessing protein phosphorylation in RMS01 cells cultured for 16 h in 10% Fetal Bovine Serum (FBS) or 0% FBS (serum starved) before exposure to vehicle or drug for 3 h and as indicated 25 ng·mL^−1^ FGF7 for the last 20 min. Quantitation of Fibroblast Growth Factor Receptor Substrate 2α (FRS2α) phosphorylation as measured by electrochemiluminescent assay in RMS01 (C) and RH41 (D) cells subject to the same treatment as in B. Results are representative of two independent experiments, except C and D, which were repeated three times. Error bars represent standard deviation with significance of differences determined by One‐Way ANOVA with Dunnett’s multiple testing correction (n.s. = not significant, **P < 0.01, ***P < 0.005, ****P < 0.001).

We sought molecular evidence of autocrine loop activity by assessing tyrosine phosphorylation levels on FGFR2 immunoprecipitated from RMS01 cells exposed to FGF7 stimulus and NVP‐BGJ398. Low basal tyrosine phosphorylation of FGFR2 in 10% FBS was modestly increased upon exposure to 25 ng·mL^−1^ FGF7 (Fig. 3A and Fig. S5A), although this was decreased by 3 h exposure to low concentrations of NVP‐BGJ398. Interestingly, we also identified tyrosine phosphorylation of a band at ~ 60 kDa (Fig. S5B), matching the size of the nuclear shorter form of FGFR2. This band increased in intensity with FGF7 stimulus but was diminished upon addition of NVP‐BGJ398, indicating that this shorter form of FGFR2 is also inhibited by pan‐FGFR inhibitors. Similarly, tyrosine phosphorylation on immunoprecipitated FGFR4 was inhibited by addition of NVP‐BGJ398, although addition of 150 ng·mL^−1^ FGF19, a common agonist of FGFR4 signaling, did not enhance basal phosphorylation (Fig. S5C,D).

The phosphorylation of signaling proteins downstream of FGFRs was then assessed to determine which were stimulated by FGF7 and which were inhibited by NVP‐BGJ398. Suppression of FGFR and ERK1/2 phosphorylation was observed with increasing concentrations of NVP‐BGJ398 in cells cultured in 10% FBS. This contrasted with no change in Signal transducer and Activator of Transcription 3 (STAT3) phosphorylation and extremely low basal levels of AKT phosphorylation (Fig. 3B). Interestingly, FGFR and ERK1/2 phosphorylation was maintained despite 16 h of serum starvation (0% FBS), indicative of autocrine loop activity, whereas STAT3 phosphorylation disappeared and AKT phosphorylation was significantly induced (Fig. 3B). Addition of exogenous FGF7 to starved cells minimally increased ERK1/2 phosphorylation but had no effect on FGFR or AKT phosphorylation, suggesting that activation of these proteins may be saturated in these conditions. Strikingly, addition of NVP‐BGJ398 suppressed phosphorylation of FGFR, ERK1/2, and AKT in a dose‐dependent manner (Fig. 3B). We observed similar results in a second fusion‐positive RMS cell line, RH41 (Fig. S5E) except we could not detect STAT3 phosphorylation and basal AKT phosphorylation was high in 10% FBS. While the latter was maintained upon starvation, it did not decrease upon exposure to drug (Fig. S5E).

Fibroblast Growth Factor Receptor Substrate 2α (FRS2α) is an adaptor protein that binds to, and is phosphorylated by, activated FGFRs leading to recruitment of further adaptor proteins that activate downstream signaling pathways, such as Mitogen Activated Protein Kinase (MAPK) and Phosphatidylinositol‐3 kinase (PI3K). Given its intimate role in transducing signals from activated FGFRs to intracellular signaling pathways, then FRS2α phosphorylation was evaluated using an electrochemiluminescent immunoassay. We observed suppression of FRS2α phosphorylation by NVP‐BGJ398 in both RMS01 and RH41 cells cultured in 10% FBS (Fig. 3C,D). In contrast to this, there was no decrease in FRS2α phosphorylation after 16 h of serum starvation (0% FBS), which together with concordant effects seen for FGFR and ERK1/2 phosphorylation strongly indicates the presence of an autocrine loop that is consistent with the known specificity of FGF7/FGFR2 (1). FGF7 stimulation of cells did increase FRS2α phosphorylation, but this was blocked with increasing concentrations of NVP‐BGJ398 (Fig. 3C,D).

### 3.5 Functional role of FGFR2 and FGF7 in FP‐RMS cells

**Fig. 4:**
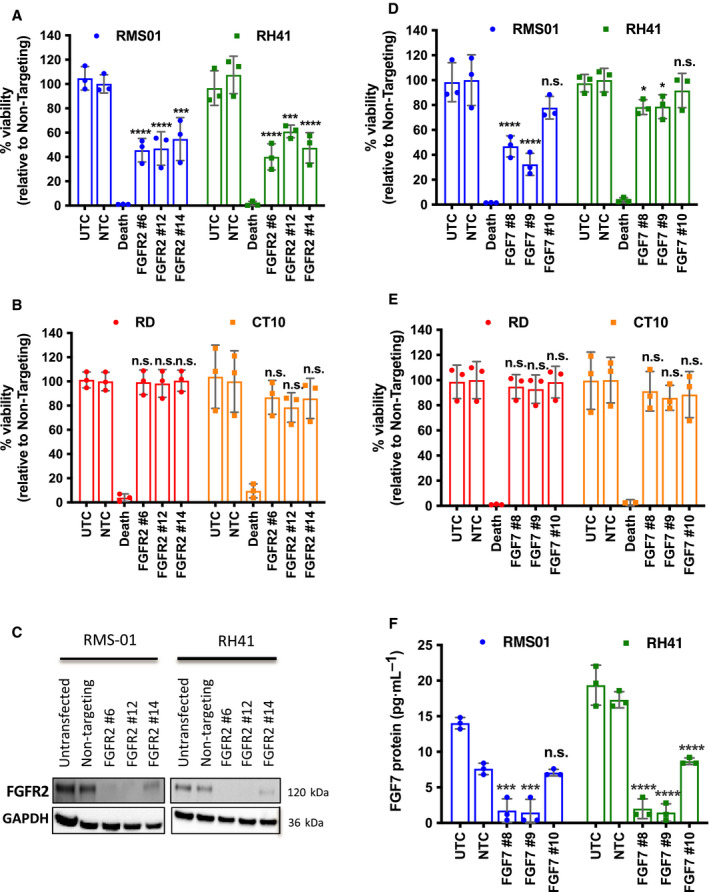
FGFR2 and FGF7 maintain FP‐RMS cell viability. Effect of 144 h short interfering RNA (siRNA) mediated FGFR2 knockdown on (A) fusion‐positive RMS (FP‐RMS) and (B) fusion‐negative RMS (FN‐RMS) cell viability. (C) Representative western blot of 72 h FGFR2 knockdown in FP‐RMS cells. Effect of 144 h siRNA mediated FGF7 knockdown on (D) FP‐RMS and (E) FN‐RMS cell viability. (F) FGF7 enzyme‐linked immunosorbent assay (ELISA) on media from FP‐RMS lines subjected to FGF7 knockdown. UTC = Untransfected control, NTC = Nontargeting control, Death = positive control siRNA that causes cell death. Results are representative of three independent experiments with error bars representing standard deviation. Significance of difference between siRNAs and NTC were tested by One‐way ANOVA with Dunnett’s multiple testing correction (n.s. = not significant, *P < 0.05, ***P < 0.005, ****P < 0.001).

Next, we tested whether FGFR2 and FGF7 have a functional role in FP‐RMS. FGFR2 knockdown with three independent small interfering RNAs (siRNAs) significantly decreased viability of the FP‐RMS cell lines RMS01 and RH41 but not the FN‐RMS lines RD and CT10 (Fig. 4A,B). Loss of FGFR2 protein in RMS01 and RH41 cells was achieved upon knockdown (Fig. 4C) with quantitative Reverse‐transcription polymerase chain reaction (qRT‐PCR) confirming reduction of *FGFR2* mRNA in all lines (Fig. S6A). *FGFR2* siRNA knockdown was thought to be specific as we observed only minimal decreases in *FGFR1* or *FGFR4* mRNA levels by qRT‐PCR (Fig. S6B,C). Despite a slight reduction in *FGFR3* mRNA levels this was not consistent between siRNAs or cell lines, suggesting that loss of viability upon FGFR2 knockdown is due to loss of FGFR2 protein.

Similarly, FGF7 knockdown using three independent siRNAs led to loss of viability in both RMS01 and RH41 cells but not in RD and CT10 cells (Fig. 4D,E). An FGF7 enzyme‐linked immunosorbent assay (ELISA) revealed that two of the three siRNAs were more effective at reducing protein upon knockdown (Fig. 4F), which was confirmed at the mRNA level by qRT‐PCR (Fig. S6D). Overall, these results demonstrate that both FGF7 and FGFR2 proteins play a role in maintaining FP‐RMS cell viability.

### 3.6 NVP‐BGJ398 inhibits the growth of FP‐RMS xenografts *in vivo*

**Fig. 5:**
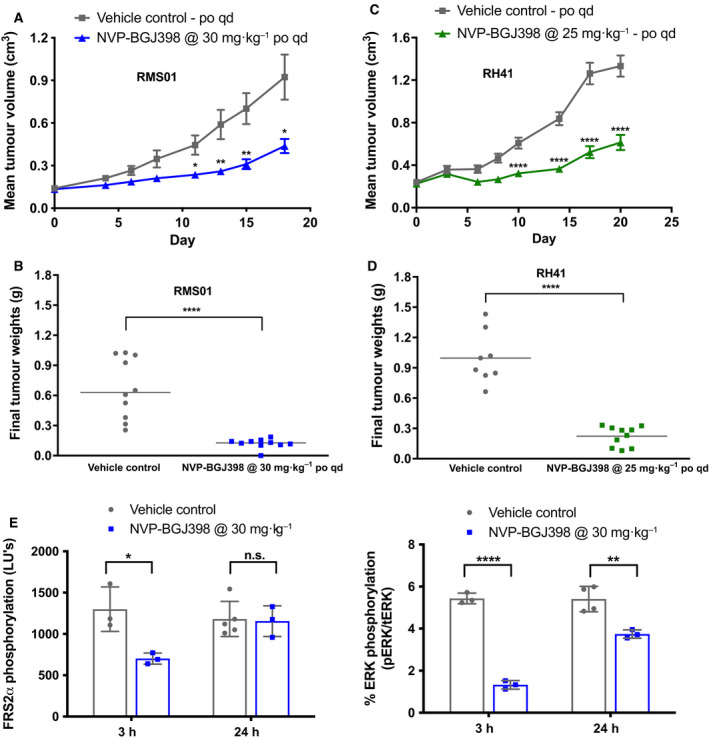
NVP‐BGJ398 inhibits the growth of FP‐RMS xenografts *in vivo*. Mean volume (A) and weight (B) of RMS01 tumor xenografts after 15 days exposure to vehicle or 30 mg·kg^−1^ NVP‐BGJ398 quaque die (q.d.). Error bars represent standard error of the mean for control (*n* = 10) and treated (*n* = 10) groups. Mean volume (C) and weight (D) of RH41 tumor xenografts after treatment with vehicle or 25 mg·kg^−1^ NVP‐BGJ398 q.d. for 20 days. Error bars represent standard error of the mean for control (*n* = 8) and treated (*n* = 10) groups. (E) Phosphorylation of Fibroblast Growth Factor Receptor Substrate 2α (FRS2α) and the ratio of phospho/total ERK1/2 in RMS01 xenografts at 3 and 24 h post dosing with 30 mg·kg^−1^ NVP‐BGJ398. Measurements were by electrochemiluminescent assay with error bars representing standard deviation of the mean for control (*n* = 3) and treated (*n* = 3) groups. Unpaired *t*‐tests with Welch’s correction were used to assess significance of differences. (n.s. = not significant, *P < 0.05, **P < 0.01, ****P < 0.001).

To assess the clinical utility of inhibiting FGFRs *in vivo,* we administered NVP‐BGJ398 daily by oral gavage to mice bearing human FP‐RMS xenografts. The mean volume and weight of RMS01 tumor xenografts were reduced by 53% (*P* = 0.0139) and 82% (*P* = 0.0003), respectively, after 15 days of oral dosing with 30 mg·kg^−1^ NVP‐BGJ398 quaque die (q.d.) compared with the vehicle control arm (Fig. 5A,B). Similarly, the mean volume and weight of RH41 tumor xenografts were reduced by 54% (*P* < 0.0001) and 78% (*P* < 0.0001), respectively, upon dosing with 25 mg·kg^−1^ q.d. NVP‐BGJ398 for 20 days compared to vehicle alone (Fig. 5C,D).

Mice dosed at either concentration suffered negligible weight loss (≤ 6%) over the course of treatment, indicating that NVP‐BGJ398 was well tolerated (Fig. S7A,B). Accumulation of NVP‐BGJ398 was observed over the course of treatment in both RMS01 and RH41 tumors compared to plasma (Fig. S7C,D) with concentrations similar to those observed in a previously published study [37]. Free plasma levels of NVP‐BGJ398 were calculated to be 2.5 nm in mice, demonstrating that antitumor effects can be achieved at doses lower than the 7 nm observed in patients from the first Phase I trial [23].

Dosing with 30 mg·kg^−1^ NVP‐BGJ398 reduced FRS2α and ERK1/2 phosphorylation by 46% (*P* = 0.02) and 76% (*P* = 0.000025), respectively, in RMS01 xenografts within 3 h compared with vehicle‐treated mice (Fig. 5E). This reflected our observations *in vitro* and with recovery of phosphorylation within 24 h *in vivo* (Fig. 5E) matched those in a previous publication [11]. IHC analysis of RH41 tumors from mice dosed with 25 mg·kg^−1^ q.d. NVP‐BGJ398 over 20 days demonstrated an average reduction of 21% (*P* < 0.0001) in Ki67 staining compared to vehicle treated mice (Fig. S7E,F). Overall, NVP‐BGJ398 was orally bioavailable, well tolerated, and reduced tumor growth as a single agent in two FP‐RMS xenograft models *in vivo*.

### 3.7 NVP‐BGJ398 is synergistic in combination with irinotecan

**Fig. 6:**
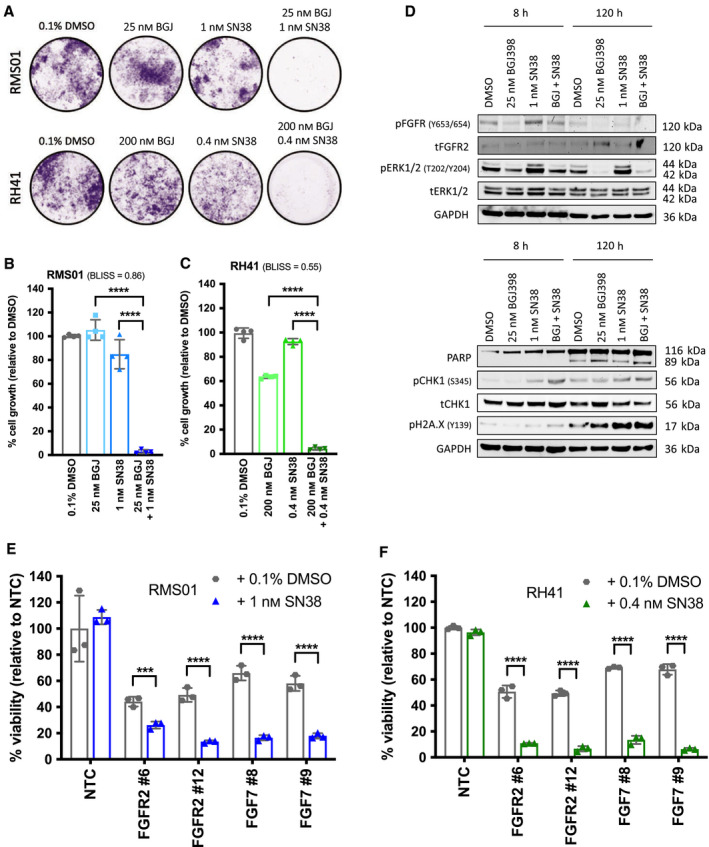
Combining NVP‐BGJ398 with SN38 is efficacious against FP‐RMS cells *in vitro*. (A) Representative images of wells from clonogenic assays in which fusion‐positive rhabdomyosarcoma (FP‐RMS) cell lines RMS01 and RH41 were exposed to the indicated drugs for 5 days before 7 days growth in media alone. Quantification of RMS01 (B) and RH41 (C) cell growth from clonogenic assays as described above. Results are representative of four independent experiments with significance of differences measured by One‐way ANOVA with Dunnett’s multiple testing correction. ****P < 0.001, Bliss scores, > 0 ≤ 1 = synergistic. (D) Representative western blots assessing protein levels in RMS01 cells *in vitro* after exposure to the indicated drugs for 8 or 120 h. Results are representative of two independent experiments. Cell viability of RMS01 (E) and RH41 (F) cells after 144 h FGFR2 or FGF7 knockdown in combination with either DMSO vehicle (gray) or SN38 (blue/green), relative to nontargeting control (NTC). Results are representative of three independent experiments with error bars representing standard deviation from the mean. Significance of differences were assessed using unpaired *t*‐tests with Welch’s correction. ***P < 0.005, ****P < 0.001.

Given the promising single agent results, we then focused on identifying an effective combination therapy, which could be incorporated into a clinical trial for RMS patients. We evaluated the efficacy of NVP‐BGJ398 when combined with the Topoisomerase I inhibitor irinotecan, a chemotherapeutic used as standard in treating RMS patients in Europe and the United States [44, 45]. An *in vitro* dose response matrix of NVP‐BGJ398 with SN38, the active metabolite of irinotecan, identified synergy between sub‐GI_50_ concentrations of each compound as calculated by the Bliss independence method [46] (Fig. S8A). Synergy was also observed when NVP‐BGJ398 was combined with vincristine, another agent used in the treatment of RMS [44, 45], or cisplatin (Fig. S8A,B). To mimic the clinical setting more closely, we used a longer term clonogenic assay with 5 days exposure to drug followed by replacement with media and 7 days further growth. Using this method, we observed that both RMS01 and RH41 cell growth was inhibited significantly more (*P* < 0.0001) when exposed to the combination of NVP‐BGJ398 and SN38 compared with exposure to either NVP‐BGJ398 or SN38 alone (Fig. 6A–C).

To understand the molecular mechanisms underpinning the effect of combining NVP‐BGJ398 and SN38, we then looked for known markers of DNA damage and replication stress by western blot. Exposure to SN38 alone or the combination of NVP‐BGJ398 and SN38 caused a modest increase in CHEK1 phosphorylation after 8 h and H2A.X phosphorylation after 120 h (5 days) indicating accrual of DNA damage over time (Fig. 6D and Fig. S9A). Whether this damage translates into initiation of cell death is not clear, as we observed an increase in cleaved PARP in RH41 cells with SN38 alone and the combination but no change in RMS01 (Fig. 6D and Fig. S9A). Intriguingly, FGFR and ERK1/2 phosphorylation was elevated after exposure to SN38 alone but combining NVP‐BGJ398 with SN38 blocked this increase at both time points (Fig. 6D and Fig. S9A). Similar results were observed in the second fusion‐positive line RH41 (Fig. S9B), indicating that FGFR signaling through ERK1/2 might be activated in response to cytotoxic stress, such as chemotherapy, as a mechanism of resistance in cells allowing them to persist in drug. Finally, we looked for evidence that FGF7 and FGFR2 specifically play a role in regulating the response of FP‐RMS cells to DNA damage. RMS01 and RH41 cells subjected to FGF7 or FGFR2 silencing suffered decreases in viability as expected, but this was significantly enhanced upon addition of low doses of SN38 that do not elicit loss of cell viability alone (Fig. 6E,F). Therefore, knockdown of either gene alone was sufficient to sensitize FP‐RMS cells to the chemotherapy over six days.

Together, these data demonstrate that FGFR signaling through ERK1/2 is elevated after exposure to SN38, which can be blocked by NVP‐BGJ398. Knockdown of FGF7 or FGFR2, or pharmacological inhibition of FGFRs, sensitizes FP‐RMS cells to SN38 *in vitro* as well as impacting on proliferation.

## 4 Discussion

Our study highlights a previously unreported FGF7‐FGFR2 signaling axis, which regulates the growth and chemosensitivity of fusion‐positive RMS cells. Reported evidence demonstrates that the PAX3‐FOXO1 fusion protein is a key driver in an aggressive subset of RMS tumors and transcriptionally targets *FGFR4*. *FGFR2* and *FGF7* have not been shown to be direct targets of PAX3‐FOXO1 although data show that *FGFR2* and *FGF7* expression levels are altered after PAX3‐FOXO1 silencing or overexpression, suggesting that PAX3‐FOXO1 may control *FGFR2*, as shown for *FGFR4* [17, 22, 47, 48]. In accordance with this, we demonstrate that both FGFR2 and FGF7 mRNA and protein are elevated in FP‐RMS cell lines compared to FN‐RMS cell lines and that *FGF7* and *FGFR2* mRNA as well as FGFR2 protein are elevated in FP‐RMS tumor samples. Intriguingly, FGFR2 was observed in the nucleus of FP‐RMS cells in patients alongside nuclear and shorter forms of the receptor in cell lines *in vitro*. Intriguingly, a shorter form of ~ 60 kDa is phosphorylated upon FGF7 stimulus but is dephosphorylated by NVP‐BGJ398. While the significance of these alternate forms of FGFR2 is not clear, nuclear forms of the receptor have been described previously in bone, prostate, and breast cancer suggesting a novel role for this receptor, which requires investigation [49, 50, 51].

Strikingly, elevated *FGF7* and *FGFR2* mRNA expression correlated with one another and with greater sensitivity to NVP‐BGJ398 in FP‐RMS cell lines *in vitro*, building on previous data, demonstrating that *FGFR4* expression in RMS cells correlates with sensitivity to FGFR inhibition [52]. We also identify RMS‐YM as the most sensitive FN‐RMS cell line tested which is likely due to the presence of *FGFR1* amplification and overexpression that we previously described in these cells [15]. Alongside *FGFR4* expression, published data demonstrates that subsets of mainly FN‐RMS cells harbor constitutively activating mutations in *FGFR4,* which can sensitize cells to FGFR inhibition. However, we demonstrate that RMS559 cells harboring the V550L *FGFR4* mutation were less sensitive to NVP‐BGJ398, which is likely explained by the presence of an activating mutation in *PTPN11* (E69K). This mutation, along with *RAS* mutations also found in FN‐RMS patients, activates the downstream MAPK pathway independently of upstream RTKs causing resistance to RTK targeted drugs [17, 21, 53]. While FP‐RMS patients rarely present with mutations in RTK downstream signal pathways, the mutation status of key signal nodes should be considered, alongside FGFR expression and mutation status, when identifying patients likely to respond to FGFR‐targeted therapy.

While autocrine loops have been characterized for PDGFs and Ephrins in FP‐RMS [54, 55], we provide novel evidence for FGFs. Our data demonstrate that FGFR intracellular signaling via the ERK/MAPkinase pathway and FGF7 secretion into culture media is maintained in FP‐RMS cells despite serum starvation. Taken together with evidence that FGF7 activates FGFR2 in FP‐RMS cells, our data strongly support this ligand‐receptor pairing forming an autocrine loop promoting FP‐RMS cell viability and survival in a similar fashion to that observed in prostate tumors and pre‐adipocytes [56, 57]. Here, we showed that knockdown of either FGF7 or FGFR2 alone and treatment with NVP‐BGJ398 decreased growth of FP‐RMS *in vitro*. However, further experiments to genetically ablate FGF7 and FGFR2 in tumor xenografts are needed to confirm the proliferative role of this signaling axis *in vivo*. Our findings also demonstrate that FGF7 and FGFR2 play a role in promoting FP‐RMS chemoresistance mirroring anti‐apoptotic and radioresistant roles shown previously for FGFR4 [19, 20, 58]. Exposure to SN38 led to an increase in FGFR signaling through ERK1/2, which is concordant with previously published data demonstrating that FGFR4 signaling through ERK1/2 promotes pro‐survival protein expression in FP‐RMS [19, 20]. We showed that the elevated FGFR signaling by SN38 through ERK1/2 can be blocked by NVP‐BGJ398 in FP‐RMS. Therefore, knockdown of FGF7 or FGFR2, or pharmacological inhibition of FGFRs, sensitizes FP‐RMS cells to SN38 *in vitro* and is consistent with FGFR signaling activating pro‐survival signals in response to DNA damage in addition to affecting proliferation. Alongside ERK1/2, another downstream candidate for such pro‐survival signaling includes mTOR enhancing FANCD2, as previously indicated in FP‐RMS [59].

Although previous studies have shown that FGFR4 signals through ERK1/2 [19, 20], signaling via STAT3 in FP‐RMS has also been reported [18, 52]. Our molecular analysis of FGFR2 did not show a change in phosphorylation of STAT3 or AKT in samples upon stimulus with FGF7 or after exposure to NVP‐BGJ398. This indicates that intracellular signaling downstream of FGFR2 and FGFR4 may be divergent in particular contexts despite showing some similarities in their biological roles [18, 19, 20]. The identification of overlapping roles for these two proteins suggests a level of functional redundancy between FGFR family members in RMS cells and emphasizes a clinical rationale to use a pan‐family FGFR inhibitor to treat FP‐RMS patients. To date, only two studies demonstrate the use of pan‐FGFR inhibitors *in vivo*. Li and colleagues used murine xenograft models to identify FGFR4 mutant, FN‐RMS cells as sensitive to ponatinib, and Crose and colleagues demonstrated antitumor effects of the tool compound PD‐173074 in FP‐RMS cells, albeit at doses that were toxic to mice.

In contrast to this, our data exemplify NVP‐BGJ398 as a pan‐family FGFR inhibitor that is orally bioavailable, well tolerated and significantly suppresses human FP‐RMS tumor xenograft growth *in vivo*. Furthermore, as our *in vitro* model demonstrates that NVP‐BGJ398 is effective in combination with irinotecan, we propose this treatment strategy has potential to improve outcomes for high‐risk fusion‐gene‐positive RMS patients.

## 5 Conclusions

FGFR signaling is involved in the development and progression of many cancer types. Previous studies of Fibroblast Growth Factor Receptors (FGFRs) in RMS have largely focused on a role for FGFR4, through activating mutations. Here, we highlight a novel autocrine loop between FGFR2 and its ligand FGF7 in RMS cells with the PAX3‐FOXO1 fusion protein. Our study demonstrates cellular dependency on this FGF7/FGFR2 autocrine loop that, in addition to FGFR4, can be targeted by NVP‐BGJ398 and is shown to reduce *in vivo* xenograft growth. We also identify synergy between SN38, the active metabolite of irinotecan, and NVP‐BGJ398 that represents an accessible potential therapeutic strategy worthy of further investigation for high‐risk fusion‐positive RMS at relapse.

## Conflict of interest

The authors declare no conflict of interest.

## Author contributions

JMS and CIM conceived the study. CIM, JS, ZSW, DGC, VK, FR, and JMS contributed to the experimental design. CIM, JS, SM, EA, CB, MG, GB, MV, AH‐B, DG, AH, and EID conducted experiments. CIM, JS, SM, EA, CB, EM, ZSW, SAG, MV, MC, EID, DGC, AK, and JMS analyzed and interpreted data. CIM prepared the manuscript, which all authors reviewed and revised.

### Peer Review

The peer review history for this article is available at https://publons.com/publon/10.1002/1878‐0261.13145.

## Supporting information


**Fig. S1**. 2D Screening of RMS cells against FGFR inhibitors.
**Fig. S2**. Correlations between RMS cell line *FGF* and *FGFR* mRNA expression along with log2 GI_50_ to NVP‐BGJ398.
**Fig. S3**. *FGFR2*, *FGF7* and *FGFR4* mRNA is highly expressed in FP‐RMS patient samples.
**Fig. S4**. FGFR2 is found in the nucleus of cells from FP‐RMS patients and cell lines.
**Fig. S5**. Effects of FGF7 and NVP‐BGJ398 on FGFR signaling in RMS cells.
**Fig. S6**. Validation of *FGFR2* and *FGF7* knockdown.
**Fig. S7**. Effects of NVP‐BGJ398 *in vivo*.
**Fig. S8**. NVP‐BGJ398 synergizes with SN38 and other DNA damaging agents in FP‐RMS cells *in vitro*.
**Fig. S9**. Molecular markers of FP‐RMS cell response to NVP‐BGJ398, SN38 or their combination.

**Table S1**. List of antibodies used for immunoblotting.
**Table S2**. Potency and selectivity profiles for FGFR inhibitors against a panel of protein targets.
**Table S3**. RMS cell line response to NVP‐BGJ398 in 2D MTS assay.

## Data Availability

The data supporting this study are available from the authors upon reasonable request. Gene expression data that support the findings of this study are openly available in EBI’s Array Express and are accessible through https://www.ebi.ac.uk/arrayexpress/experiments/E‐TABM‐1202/ as well as in NCBI’s Gene Expression Omnibus through https://www.ncbi.nlm.nih.gov/geo/query/acc.cgi?acc=GSE92689. RNA‐Seq data that support the findings of this study are available through the Khan laboratory at https://omics‐oncogenomics.ccr.cancer.gov/cgi‐bin/JK.

## References

[1] Zhang X , Ibrahimi OA , Olsen SK , Umemori H , Mohammadi M , Ornitz DM . Receptor specificity of the fibroblast growth factor family. The complete mammalian FGF family. J Biol Chem. 2006;23:15694–700. 10.1074/jbc.M601252200 PMC2080618 16597617

[2] Turner N , Grose R . Fibroblast growth factor signalling: from development to cancer. Nat Rev Cancer. 2010;2:116–29. 10.1038/nrc2780 20094046

[3] Itoh N , Ornitz DM . Fibroblast growth factors: from molecular evolution to roles in development, metabolism and disease. J Biochem. 2011;2:121–30. 10.1093/jb/mvq121 PMC3106964 20940169

[4] Oladipupo SS , Smith C , Santeford A , Park C , Sene A , Wiley LA , et al. Endothelial cell FGF signaling is required for injury response but not for vascular homeostasis. Proc Natl Acad Sci USA. 2014;111:13379–84. 25139991 10.1073/pnas.1324235111 PMC4169958

[5] Dell'Era P , Ronca R , Coco L , Nicoli S , Metra M , Presta M . Fibroblast growth factor receptor‐1 is essential for in vitro cardiomyocyte development. Circ Res. 2003;5:414–20. 10.1161/01.RES.0000089460.12061.E1 12893744

[6] White AC , Xu J , Yin Y , Smith C , Schmid G , Ornitz DM . FGF9 and SHH signaling coordinate lung growth and development through regulation of distinct mesenchymal domains. Development. 2006;8:1507–17. 10.1242/dev.02313 16540513

[7] Draper BW , Stock DW , Kimmel CB . Zebrafish fgf24 functions with fgf8 to promote posterior mesodermal development. Development. 2003;19:4639–54. 10.1242/dev.00671 12925590

[8] Flanagan‐Steet H , Hannon K , McAvoy MJ , Hullinger R , Olwin BB . Loss of FGF receptor 1 signaling reduces skeletal muscle mass and disrupts myofiber organization in the developing limb. Dev Biol. 2000;1:21–37. 10.1006/dbio.1999.9535 10644408

[9] Parakati R , DiMario JX . Repression of myoblast proliferation and fibroblast growth factor receptor 1 promoter activity by KLF10 protein. J Biol Chem. 2013;19:13876–84. 10.1074/jbc.M113.457648 PMC3650423 23569208

[10] Helsten T , Elkin S , Arthur E , Tomson BN , Carter J , Kurzrock R . The FGFR landscape in cancer: analysis of 4,853 tumors by next‐generation sequencing. Clin Cancer Res. 2016;1:259–67. 10.1158/1078-0432.CCR-14-3212 26373574

[11] Guagnano V , Kauffmann A , Wohrle S , Stamm C , Ito M , Barys L , et al. FGFR genetic alterations predict for sensitivity to NVP‐BGJ398, a selective pan‐FGFR inhibitor. Cancer Discov. 2012;12:1118–33. 10.1158/2159-8290.CD-12-0210 23002168

[12] Malchers F , Dietlein F , Schottle J , Lu X , Nogova L , Albus K , et al. Cell‐autonomous and non‐cell‐autonomous mechanisms of transformation by amplified FGFR1 in lung cancer. Cancer Discov. 2013;4:246‐57. 24302556 10.1158/2159-8290.CD-13-0323

[13] Mathur A , Ware C , Davis L , Gazdar A , Pan BS , Lutterbach B . FGFR2 is amplified in the NCI‐H716 colorectal cancer cell line and is required for growth and survival. PLoS One. 2014;6:e98515. 10.1371/journal.pone.0098515 PMC4072591 24968263

[14] Goldstein M , Meller I , Issakov J , Orr‐Urtreger A . Novel genes implicated in embryonal, alveolar, and pleomorphic rhabdomyosarcoma: a cytogenetic and molecular analysis of primary tumors. Neoplasia. 2006;5:332–43. 10.1593/neo.05829 PMC1592451 16790082

[15] Missiaglia E , Selfe J , Hamdi M , Williamson D , Schaaf G , Fang C , et al. Genomic imbalances in rhabdomyosarcoma cell lines affect expression of genes frequently altered in primary tumors: an approach to identify candidate genes involved in tumor development. Genes Chromosomes Cancer. 2009;6:455–67. 10.1002/gcc.20655 19235922

[16] Cao L , Yu Y , Bilke S , Walker RL , Mayeenuddin LH , Azorsa DO , et al. Genome‐wide identification of PAX3‐FKHR binding sites in rhabdomyosarcoma reveals candidate target genes important for development and cancer. Cancer Res. 2010;16:6497–508. 10.1158/0008-5472.CAN-10-0582 PMC2922412 20663909

[17] Shern JF , Chen L , Chmielecki J , Wei JS , Patidar R , Rosenberg M , et al. Comprehensive genomic analysis of rhabdomyosarcoma reveals a landscape of alterations affecting a common genetic axis in fusion‐positive and fusion‐negative tumors. Cancer Discov. 2014;2:216–31. 10.1158/2159-8290.CD-13-0639 PMC4462130 24436047

[18] Taylor JG , Cheuk AT , Tsang PS , Chung JY , Song YK , Desai K , et al. Identification of FGFR4‐activating mutations in human rhabdomyosarcomas that promote metastasis in xenotransplanted models. J Clin Invest. 2009;11:3395–407. 10.1172/JCI39703 PMC2769177 19809159

[19] Crose LE , Etheridge KT , Chen C , Belyea B , Talbot LJ , Bentley RC , et al. FGFR4 blockade exerts distinct antitumorigenic effects in human embryonal versus alveolar rhabdomyosarcoma. Clin Cancer Res. 2012;14:3780–90. 10.1158/1078-0432.CCR-10-3063 PMC3713717 22648271

[20] Wachtel M , Rakic J , Okoniewski M , Bode P , Niggli F , Schafer BW . FGFR4 signaling couples to Bim and not Bmf to discriminate subsets of alveolar rhabdomyosarcoma cells. Int J Cancer. 2014;7:1543–52. 10.1002/ijc.28800 24550147

[21] Shukla N , Ameur N , Yilmaz I , Nafa K , Lau CY , Marchetti A , et al. Oncogene mutation profiling of pediatric solid tumors reveals significant subsets of embryonal rhabdomyosarcoma and neuroblastoma with mutated genes in growth signaling pathways. Clin Cancer Res. 2012;3:748–57. 10.1158/1078-0432.CCR-11-2056 PMC3271129 22142829

[22] Monsma DJ , Cherba DM , Richardson PJ , Vance S , Rangarajan S , Dylewski D , et al. Using a rhabdomyosarcoma patient‐derived xenograft to examine precision medicine approaches and model acquired resistance. Pediatr Blood Cancer. 2014;9:1570–7. 10.1002/pbc.25039 24687871

[23] Nogova L , Sequist LV , Perez Garcia JM , Andre F , Delord JP , Hidalgo M , et al. Evaluation of BGJ398, a fibroblast growth factor receptor 1–3 kinase inhibitor, in patients with advanced solid tumors harboring genetic alterations in fibroblast growth factor receptors: results of a global phase I, dose‐escalation and dose‐expansion study. J Clin Oncol. 2016;35:157‐65. 27870574 10.1200/JCO.2016.67.2048 PMC6865065

[24] Cortes JE , Kim DW , Pinilla‐Ibarz J , le Coutre P , Paquette R , Chuah C , et al. A phase 2 trial of ponatinib in Philadelphia chromosome‐positive leukemias. N Engl J Med. 2013;19:1783–96. 10.1056/NEJMoa1306494 PMC3886799 24180494

[25] Kim KB , Chesney J , Robinson D , Gardner H , Shi MM , Kirkwood JM . Phase I/II and pharmacodynamic study of dovitinib (TKI258), an inhibitor of fibroblast growth factor receptors and VEGF receptors, in patients with advanced melanoma. Clin Cancer Res. 2011;23:7451–61. 10.1158/1078-0432.CCR-11-1747 21976540

[26] Okamoto I , Kaneda H , Satoh T , Okamoto W , Miyazaki M , Morinaga R , et al. Phase I safety, pharmacokinetic, and biomarker study of BIBF 1120, an oral triple tyrosine kinase inhibitor in patients with advanced solid tumors. Mol Cancer Ther. 2010;10:2825–33. 10.1158/1535-7163.MCT-10-0379 20688946

[27] Hinson AR , Jones R , Crose LE , Belyea BC , Barr FG , Linardic CM . Human rhabdomyosarcoma cell lines for rhabdomyosarcoma research: utility and pitfalls. Front Oncol. 2013;3:183. 23882450 10.3389/fonc.2013.00183 PMC3713458

[28] Milton CK , Self AJ , Clarke PA , Banerji U , Piccioni F , Root DE , et al. A genome‐scale CRISPR screen identifies the ERBB and mTOR signaling networks as key determinants of response to PI3K inhibition in pancreatic cancer. Mol Cancer Ther. 2020;7:1423–35. 10.1158/1535-7163.MCT-19-1131 PMC7611271 32371585

[29] Williamson D , Missiaglia E , de Reynies A , Pierron G , Thuille B , Palenzuela G , et al. Fusion gene‐negative alveolar rhabdomyosarcoma is clinically and molecularly indistinguishable from embryonal rhabdomyosarcoma. J Clin Oncol. 2010;13:2151–8. 10.1200/JCO.2009.26.3814 20351326

[30] Selfe J , Goddard NC , McIntyre A , Taylor KR , Renshaw J , Popov SD , et al. IGF1R signalling in testicular germ cell tumour cells impacts on cell survival and acquired cisplatin resistance. J Pathol. 2018;2:242–53. 10.1002/path.5008 PMC5817239 29160922

[31] Workman P , Aboagye EO , Balkwill F , Balmain A , Bruder G , Chaplin DJ , et al. Guidelines for the welfare and use of animals in cancer research. Br J Cancer. 2010;11:1555–77. 10.1038/sj.bjc.6605642 PMC2883160 20502460

[32] Raynaud FI , Eccles S , Clarke PA , Hayes A , Nutley B , Alix S , et al. Pharmacologic characterization of a potent inhibitor of class I phosphatidylinositide 3‐kinases. Cancer Res. 2007;12:5840–50. 10.1158/0008-5472.CAN-06-4615 17575152

[33] Selfe J , Olmos D , Al‐Saadi R , Thway K , Chisholm J , Kelsey A , et al. Impact of fusion gene status versus histology on risk‐stratification for rhabdomyosarcoma: retrospective analyses of patients on UK trials. Pediatr Blood Cancer. 2016;64:e26386. 10.1002/pbc.26386 28035744

[34] Moslehi JJ , Deininger M . Tyrosine kinase inhibitor‐associated cardiovascular toxicity in chronic myeloid leukemia. J Clin Oncol. 2015;35:4210–8. 10.1200/JCO.2015.62.4718 PMC4658454 26371140

[35] Paech F , Mingard C , Grunig D , Abegg VF , Bouitbir J , Krahenbuhl S . Mechanisms of mitochondrial toxicity of the kinase inhibitors ponatinib, regorafenib and sorafenib in human hepatic HepG2 cells. Toxicology. 2018;395:34–44. 29341879 10.1016/j.tox.2018.01.005

[36] Medeiros BC , Possick J , Fradley M . Cardiovascular, pulmonary, and metabolic toxicities complicating tyrosine kinase inhibitor therapy in chronic myeloid leukemia: strategies for monitoring, detecting, and managing. Blood Rev. 2018;4:289–99. 10.1016/j.blre.2018.01.004 29454474

[37] Guagnano V , Furet P , Spanka C , Bordas V , Le Douget M , Stamm C , et al. Discovery of 3‐(2,6‐dichloro‐3,5‐dimethoxy‐phenyl)‐1‐{6‐[4‐(4‐ethyl‐piperazin‐1‐yl)‐phenylamin o]‐pyrimidin‐4‐yl}‐1‐methyl‐urea (NVP‐BGJ398), a potent and selective inhibitor of the fibroblast growth factor receptor family of receptor tyrosine kinase. J Med Chem. 2011;20:7066–83. 10.1021/jm2006222 21936542

[38] O'Hare T , Shakespeare WC , Zhu X , Eide CA , Rivera VM , Wang F , et al. AP24534, a pan‐BCR‐ABL inhibitor for chronic myeloid leukemia, potently inhibits the T315I mutant and overcomes mutation‐based resistance. Cancer Cell. 2009;5:401–12. 10.1016/j.ccr.2009.09.028 PMC2804470 19878872

[39] Lee SH , Lopes de Menezes D , Vora J , Harris A , Ye H , Nordahl L , et al. In vivo target modulation and biological activity of CHIR‐258, a multitargeted growth factor receptor kinase inhibitor, in colon cancer models. Clin Cancer Res. 2005;10:3633–41. 10.1158/1078-0432.CCR-04-2129 15897558

[40] Hilberg F , Roth GJ , Krssak M , Kautschitsch S , Sommergruber W , Tontsch‐Grunt U , et al. BIBF 1120: triple angiokinase inhibitor with sustained receptor blockade and good antitumor efficacy. Cancer Res. 2008;12:4774–82. 10.1158/0008-5472.CAN-07-6307 18559524

[41] LaRochelle JR , Fodor M , Xu X , Durzynska I , Fan L , Stams T , et al. Structural and functional consequences of three cancer‐associated mutations of the oncogenic phosphatase SHP2. Biochemistry. 2016;15:2269–77. 10.1021/acs.biochem.5b01287 PMC4900891 27030275

[42] Prahallad A , Heynen GJ , Germano G , Willems SM , Evers B , Vecchione L , et al. PTPN11 is a central node in intrinsic and acquired resistance to targeted cancer drugs. Cell Rep. 2015;12:1978–85. 26365186 10.1016/j.celrep.2015.08.037

[43] Davicioni E , Finckenstein FG , Shahbazian V , Buckley JD , Triche TJ , Anderson MJ . Identification of a PAX‐FKHR gene expression signature that defines molecular classes and determines the prognosis of alveolar rhabdomyosarcomas. Cancer Res. 2006;14:6936–46. 10.1158/0008-5472.CAN-05-4578 16849537

[44] Defachelles AS , Bogart E , Casanova M , Merks H , Bisogno G , Calareso G , et al. Randomized phase 2 trial of the combination of vincristine and irinotecan with or without temozolomide, in children and adults with refractory or relapsed rhabdomyosarcoma (RMS). J Clin Oncol. 2019;37(15_suppl):10000. 10.1200/JCO.21.00124 34343032

[45] Hawkins DS , Chi YY , Anderson JR , Tian J , Arndt CAS , Bomgaars L , et al. Addition of vincristine and irinotecan to vincristine, dactinomycin, and cyclophosphamide does not improve outcome for intermediate‐risk rhabdomyosarcoma: a report from the children's oncology group. J Clin Oncol. 2018;27:2770–7. 10.1200/JCO.2018.77.9694 PMC6145831 30091945

[46] Greco WR , Bravo G , Parsons JC . The search for synergy: a critical review from a response surface perspective. Pharmacol Rev. 1995;2:331–85. 7568331

[47] Gryder BE , Yohe ME , Chou H‐C , Zhang X , Marques J , Wachtel M , et al. PAX3–FOXO1 establishes myogenic super enhancers and confers BET bromodomain vulnerability. Cancer Discov. 2017;7:884–99. 28446439 10.1158/2159-8290.CD-16-1297 PMC7802885

[48] Ebauer M , Wachtel M , Niggli FK , Schafer BW . Comparative expression profiling identifies an in vivo target gene signature with TFAP2B as a mediator of the survival function of PAX3/FKHR. Oncogene. 2007;51:7267–81. 10.1038/sj.onc.1210525 17525748

[49] Cerliani JP , Guillardoy T , Giulianelli S , Vaque JP , Gutkind JS , Vanzulli SI , et al. Interaction between FGFR‐2, STAT5, and progesterone receptors in breast cancer. Cancer Res. 2011;10:3720–31. 10.1158/0008-5472.CAN-10-3074 21464042

[50] Salva JE , Roberts RR , Stucky TS , Merrill AE . Nuclear FGFR2 regulates musculoskeletal integration within the developing limb. Dev Dyn. 2019;3:233–46. 10.1002/dvdy.9 PMC6474847 30620790

[51] Lee JE , Shin SH , Shin HW , Chun YS , Park JW . Nuclear FGFR2 negatively regulates hypoxia‐induced cell invasion in prostate cancer by interacting with HIF‐1 and HIF‐2. Sci Rep. 2019;1:3480. 10.1038/s41598-019-39843-6 PMC6401139 30837551

[52] Li SQ , Cheuk AT , Shern JF , Song YK , Hurd L , Liao H , et al. Targeting wild‐type and mutationally activated FGFR4 in rhabdomyosarcoma with the inhibitor ponatinib (AP24534). PLoS One. 2013;10:e76551. 10.1371/journal.pone.0076551 PMC3790700 24124571

[53] Chen Y , Takita J , Hiwatari M , Igarashi T , Hanada R , Kikuchi A , et al. Mutations of the PTPN11 and RAS genes in rhabdomyosarcoma and pediatric hematological malignancies. Genes Chromosomes Cancer. 2006;6:583–91. 10.1002/gcc.20322 16518851

[54] Ehnman M , Missiaglia E , Folestad E , Selfe J , Strell C , Thway K , et al. Distinct effects of ligand‐induced PDGFRalpha and PDGFRbeta signaling in the human rhabdomyosarcoma tumor cell and stroma cell compartments. Cancer Res. 2013;7:2139–49. 10.1158/0008-5472.CAN-12-1646 PMC3672973 23338608

[55] Aslam MI , Abraham J , Mansoor A , Druker BJ , Tyner JW , Keller C . PDGFRbeta reverses EphB4 signaling in alveolar rhabdomyosarcoma. Proc Natl Acad Sci USA. 2014;111:6383‐8. 24733895 10.1073/pnas.1403608111 PMC4035936

[56] Huang YW , Wang LS , Chang HL , Ye W , Shu S , Sugimoto Y , et al. Effect of keratinocyte growth factor on cell viability in primary cultured human prostate cancer stromal cells. J Steroid Biochem Mol Biol. 2006;1–3:24–33. 10.1016/j.jsbmb.2006.03.005 16854582

[57] Zhang T , Guan H , Yang K . Keratinocyte growth factor promotes preadipocyte proliferation via an autocrine mechanism. J Cell Biochem. 2010;4:737–46. 10.1002/jcb.22452 20069574

[58] Benzina S , Pitaval A , Lemercier C , Lustremant C , Frouin V , Wu N , et al. A kinome‐targeted RNAi‐based screen links FGF signaling to H2AX phosphorylation in response to radiation. Cell Mol Life Sci. 2015;18:3559–73. 10.1007/s00018-015-1901-7 PMC4548013 25894690

[59] Shen C , Oswald D , Phelps D , Cam H , Pelloski CE , Pang Q , et al. Regulation of FANCD2 by the mTOR pathway contributes to the resistance of cancer cells to DNA double‐strand breaks. Cancer Res. 2013;11:3393–401. 10.1158/0008-5472.CAN-12-4282 PMC3674187 23633493

