# Supplementary material for: FGF7–FGFR2 autocrine signaling increases growth and chemoresistance of fusion‐positive rhabdomyosarcomas
Source: Mol Oncol. 2021 Dec 18;16(6):1272–89. doi: 10.1002/1878-0261.13145 (PMC8936514; doi:10.1002/1878-0261.13145)
Supplement: Supplementary file 1 — Fig. S1. 2D Screening of RMS cells against FGFR inhibitors. Fig. S2. Correlations between RMS cell line FGF and FGFR mRNA expression along with log2 GI50 to NVP‐BGJ398. Fig. S3. FGFR2, FGF7 and FGFR4 mRNA is highly expressed in FP‐RMS patient samples. Fig. S4. FGFR2 is found in the nucleus of cells from FP‐RMS patients and cell lines. Fig. S5. Effects of FGF7 and NVP‐BGJ398 on FGFR signaling in RMS cells. Fig. S6. Validation of FGFR2 and FGF7 knockdown. Fig. S7. Effects of NVP‐BGJ398 in vivo. Fig. S8. NVP‐BGJ398 synergizes with SN38 and other DNA damaging agents in FP‐RMS cells in vitro. Fig. S9. Molecular markers of FP‐RMS cell response to NVP‐BGJ398, SN38 or their combination. [file MOL2-16-1272-s001.pdf]

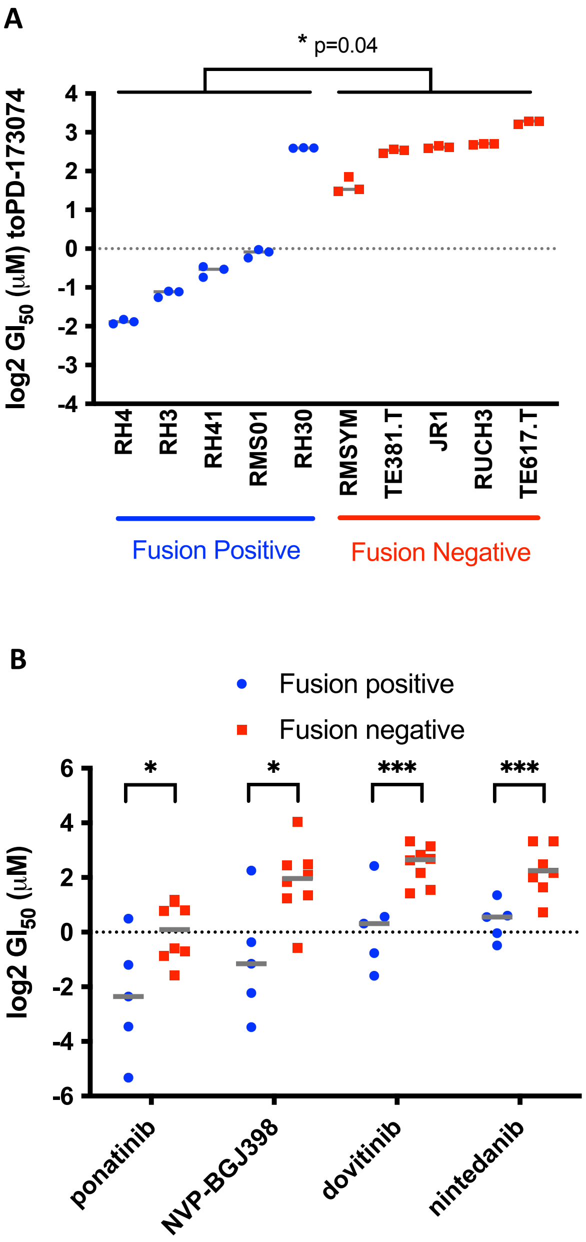

Supplementary Figure 1. 2D screening of RMS cells against FGFR inhibitors. (**A**) Waterfall plot of log2 GI_{50}s of PAX3-FOXO1 fusion positive (FP, blue) and fusion negative (FN, red) RMS cell lines to PD-173074 after 72 hours in 2D culture. (**B**) Dot plot of 2D cultured FP-RMS (blue) and FN-RMS (red) cell line log2 GI_{50}s for each of the compounds shown. Viability was measured after 72hrs by MTS. Results are representative of three independent experiments with error bars representing standard deviation. Significance of differences were assessed using unpaired t-tests with Welch's correction. *= p<0.05, ***= p<0.005.

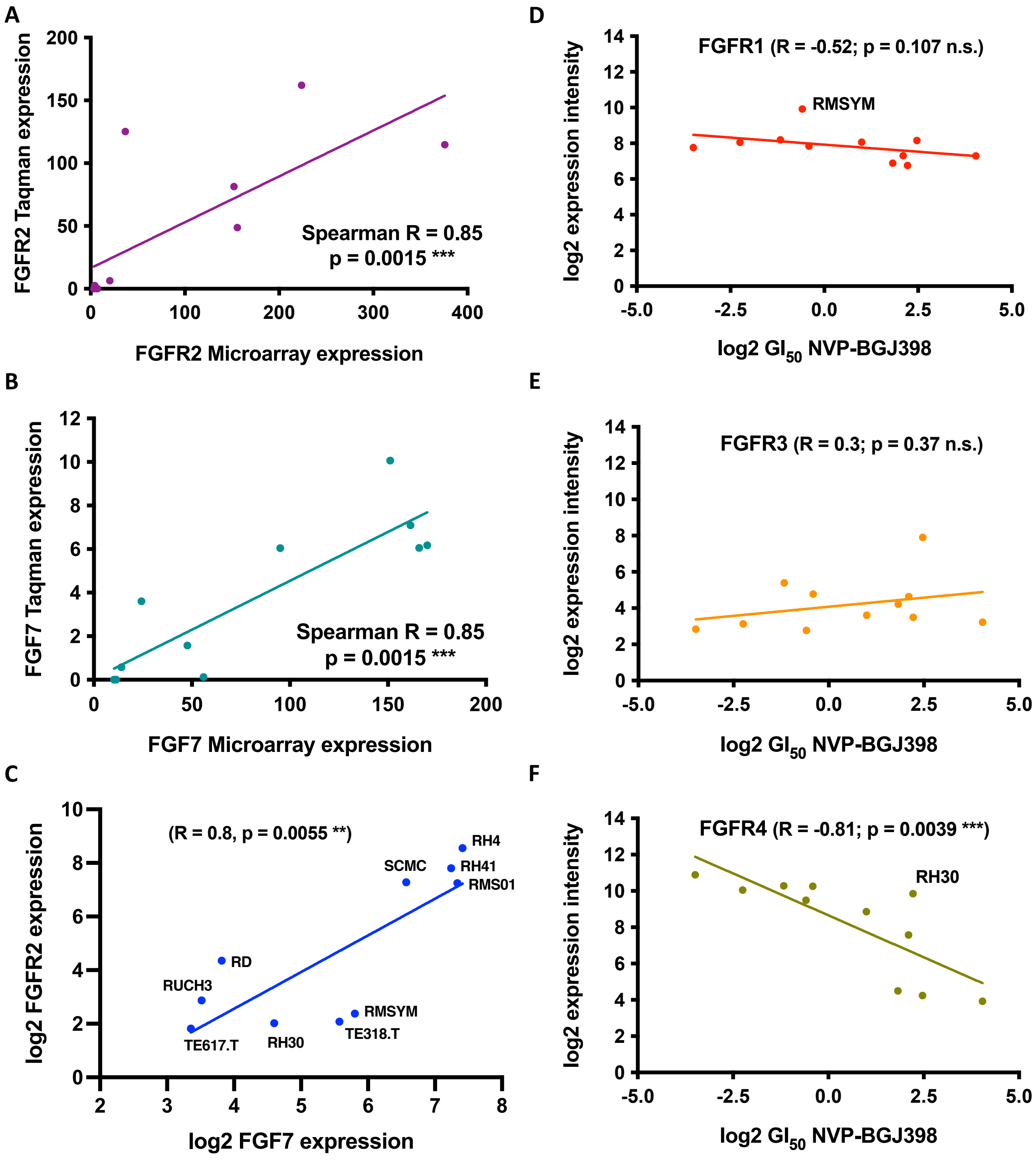

Supplementary Figure 2. Correlation between RMS cell line *FGFR* mRNA expression and log2 GI$_{50}$ for NVP-BGJ398. Scatter plots showing the correlation between Affymetrix microarray and qRT-PCR (Taqman) measurements of *FGFR2* (**A**) (plum) and *FGF7* (**B**) (teal) mRNA in RMS cell lines. Taqman expression is fold change relative to normal skeletal muscle and microarray expression is gcrma normalised intensity. (**C**) Correlation between log2 *FGFR2* and *FGF7* mRNA expression as measured by Affymetrix microarray for RMS cell lines. Scatter plots of *FGFR1* (**D**), *FGFR3* (**E**) and *FGFR4* (**F**) expression as measured by Affymetrix microarray, with log2 GI$_{50}$ for NVP-BGJ398. The line of best fit, spearman r and associated p values are indicated on each graph.

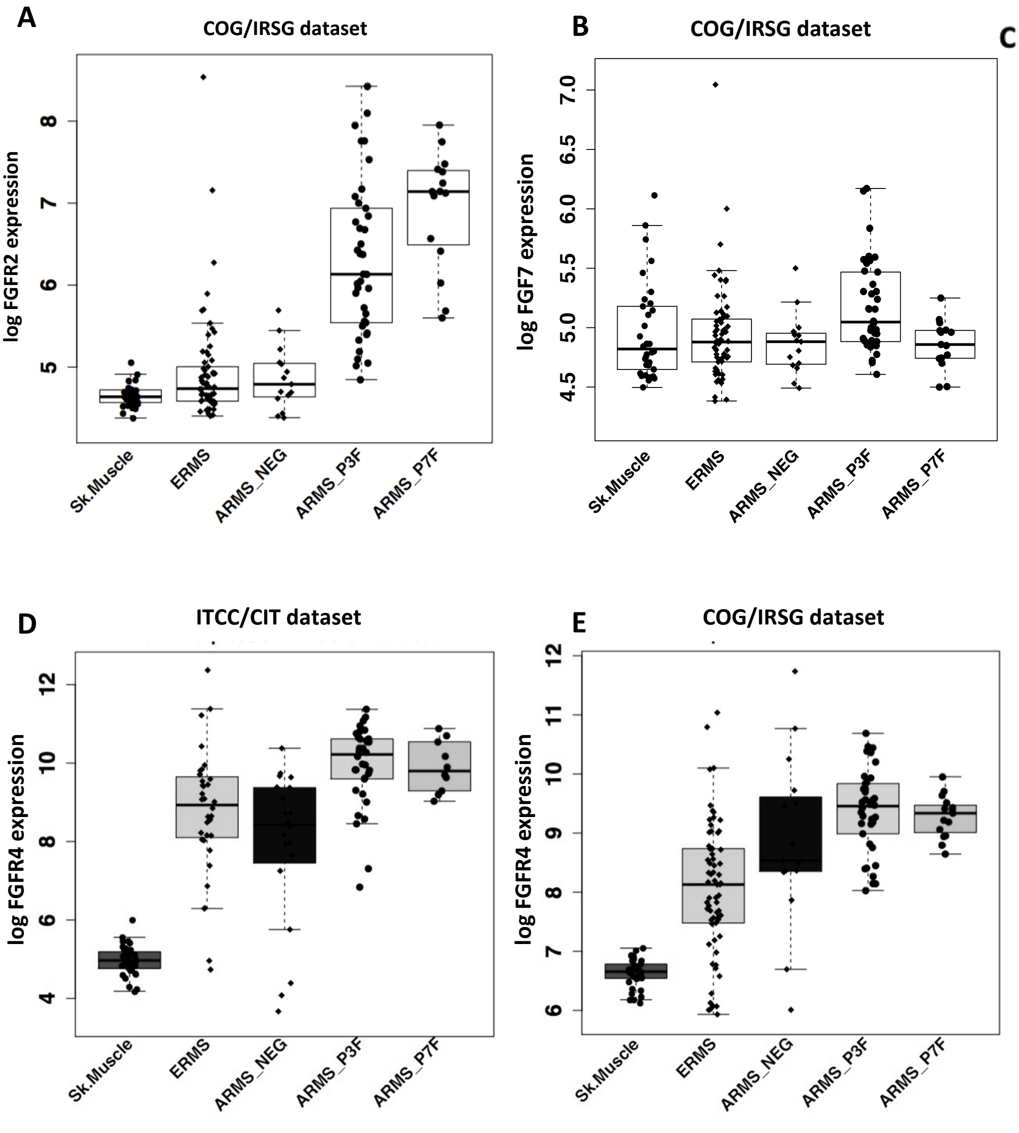

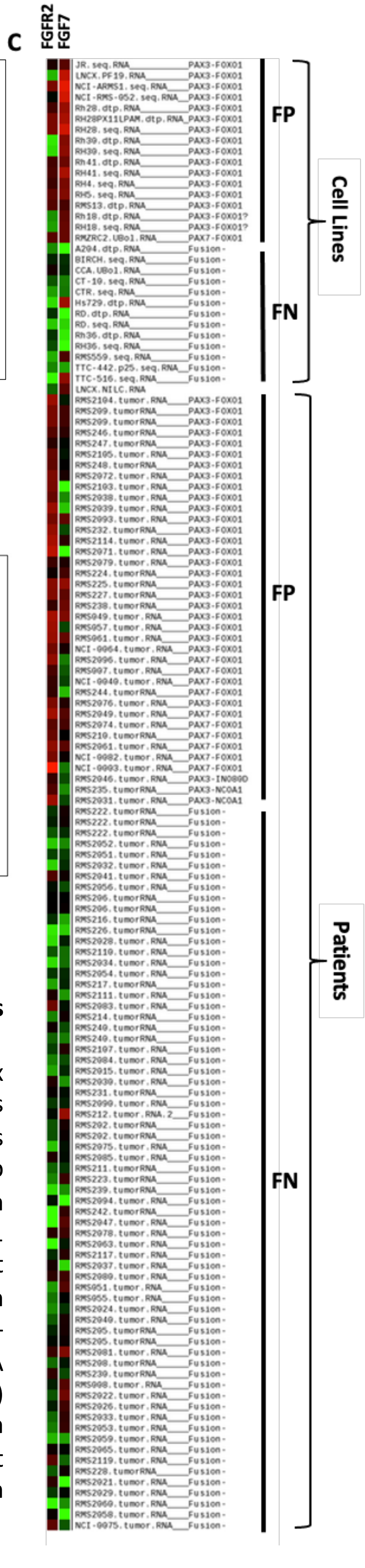

Supplementary Figure 3. *FGFR2, FGF7 and FGFR4* mRNA expression is highly expressed in FP-RMS patient samples

FGFR2 (**A**) and *FGF7* (**B**) mRNA expression, as measured by Affymetrix microarray, is significantly greater in FP-RMS (P3F; PAX3-FOXO1) patients compared to FN-RMS (ERMS and ARMS-NEG) from the Children's Oncology Group/Intergroup Rhabdomyosarcoma Study Group (COG/IRSG) cohort. (n=139 patients) Wilcoxon rank sum test for both genes is p<0.001. (**C**) RNASeq data (from Shern, J.F. *et al* 4(2):216-31 2014) validates findings from Affymetrix microarrays that FP-RMS patient samples and cell lines harbour greater *FGFR2* and *FGF7* expression compared to FN-RMS samples and cell lines. (n=29 FP-RMS and 51 FN-RMS patients). (**D** and **E**) *FGFR4* mRNA expression as measured by cDNA microarray from patient samples in the ITCC/CIT (**D**) and COG/IRSG (**E**) cohorts. All RMS samples have higher *FGFR4* expression compared with normal skeletal muscle (Wilcoxon rank sum test, P=<0.001) but is highest in fusion positive RMS (Wilcoxon rank sum test, P=<0.001) in both cohorts.

A

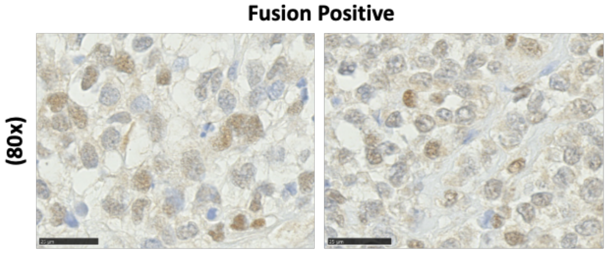

B

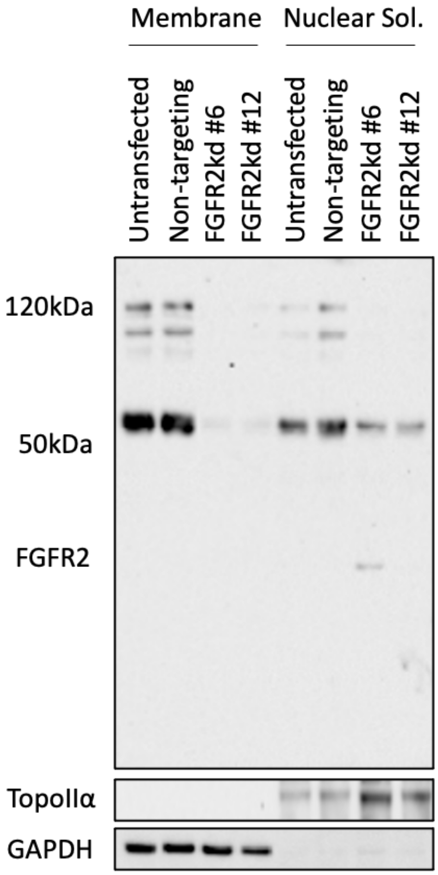

Supplementary Figure 4. FGFR2 is found in the nucleus of cells from FP-RMS patients and cell lines. (**A**) Representative images of nuclear FGFR2 staining in FP-RMS patients at 80x magnification. Scale bars represent 25µm. Image is representative of 12 FP-RMS samples. (**B**) Representative Western blot of FGFR2 in different subcellular fractions from RMS01 cells subjected to FGFR2 kd for 72 hours. TopoIIα and GAPDH were used as nuclear and membrane fraction specific markers respectively. Results are representative of two independent experiments.

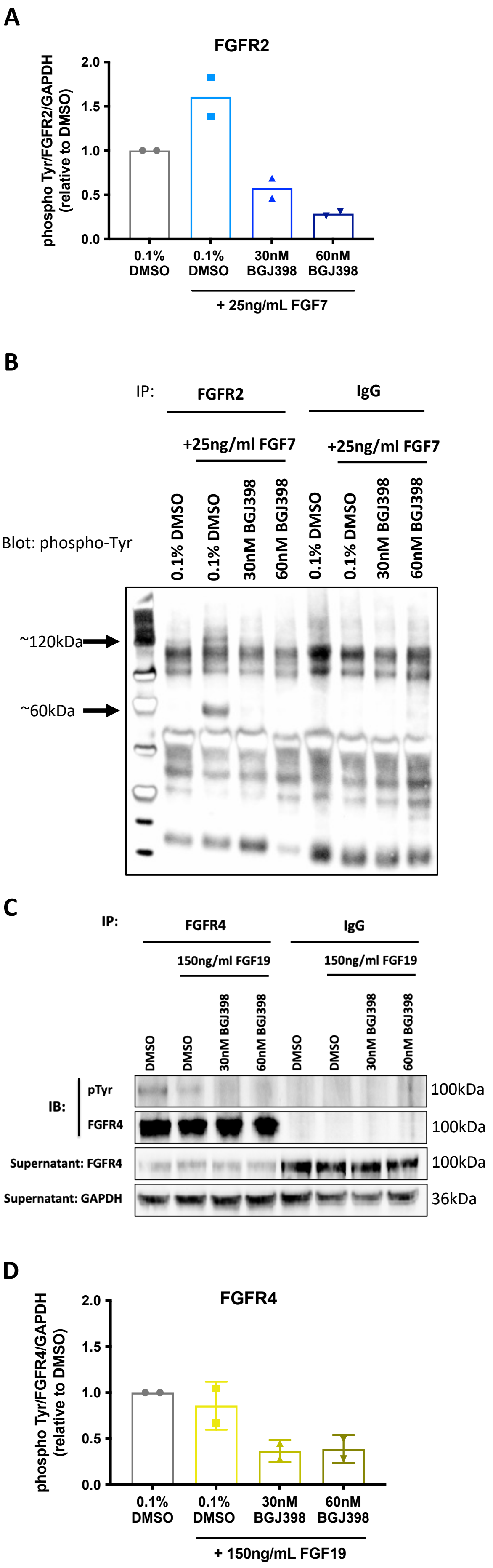

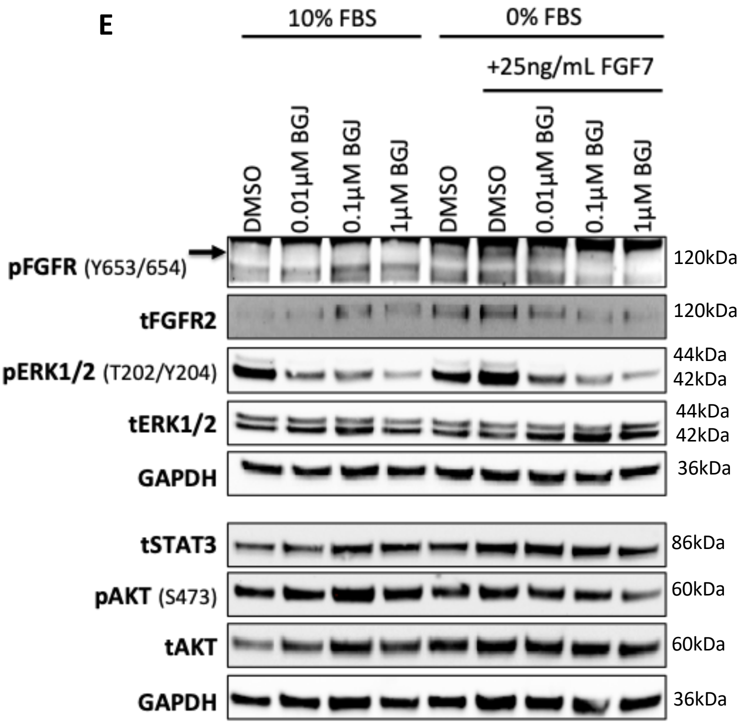

Supplementary Figure 5. Effects of FGF7 and NVP-BGJ398 on FGFR signalling in RMS cells.
(**A**) Quantitation of FGFR2 tyrosine phosphorylation in RMS01 cells exposed to FGF7 and NVP-BGJ398 (from western blot in Figure 3A). The ratio of tyrosine phosphorylation to total FGFR2 pulled down was normalised to the ratio of total FGFR2 pulled down to GAPDH. Resulting ratios for each treatment were then normalised to unstimulated cells with DMSO alone. (**B**) Full blot of tyrosine phosphorylation on FGFR2 immunoprecipitated from RMS01 cells exposed to DMSO or NVP-BGJ398 for 3 hours and 25ng/mL FGF7 for the final 20 minutes. Bands indicate long (~120kDa) and short (~60kDa) forms of FGFR2 (**C**) IP of FGFR4 from RMS01 cells exposed to DMSO or NVP-BGJ398 for 3 hours and 150ng/mL FGF19 for the final 20 minutes. (**D**) Quantification of blot B. Normalisation was conducted as described in A for FGFR2. (**E**) RH41 cells were cultured in 10% FBS or starved (0% FBS) for 16 hours before exposure to a range of concentrations of NVP-BGJ398 for 3 hours. Cells in 0% FBS were exposed to 25ng/mL FGF7 for the last 20 minutes of drug treatment before being lysed, and the phosphorylation status of several signalling proteins assessed by western blot. The band corresponding to phosphorylation of FGFR is indicated by the arrow in the uppermost panel. N=2

A

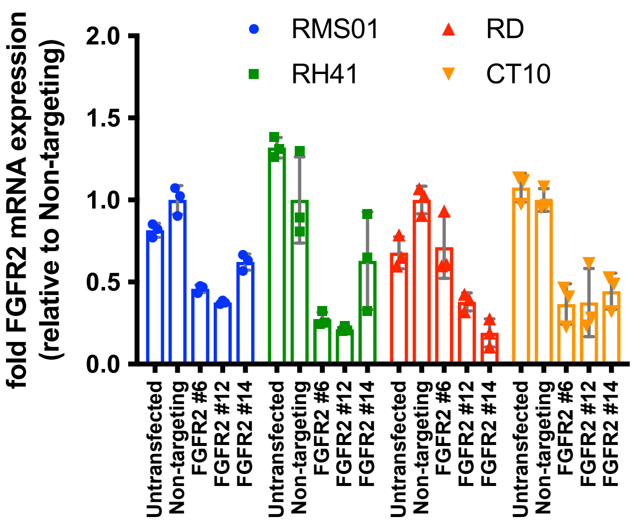

	RMS01	RH41	RD	CT10
NTC vs FGFR2 #6	****	***	*	****
NTC vs FGFR2 #12	****	***	****	****
NTC vs FGFR2 #14	****	n.s.	****	***

C

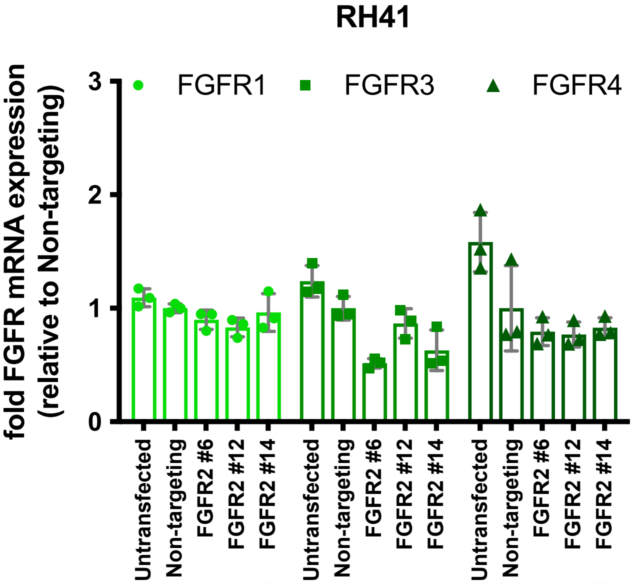

	FGFR1	FGFR3	FGFR4
NTC vs FGFR2 #6	n.s.	***	n.s.
NTC vs FGFR2 #12	n.s.	n.s.	n.s.
NTC vs FGFR2 #14	n.s.	*	n.s.

B

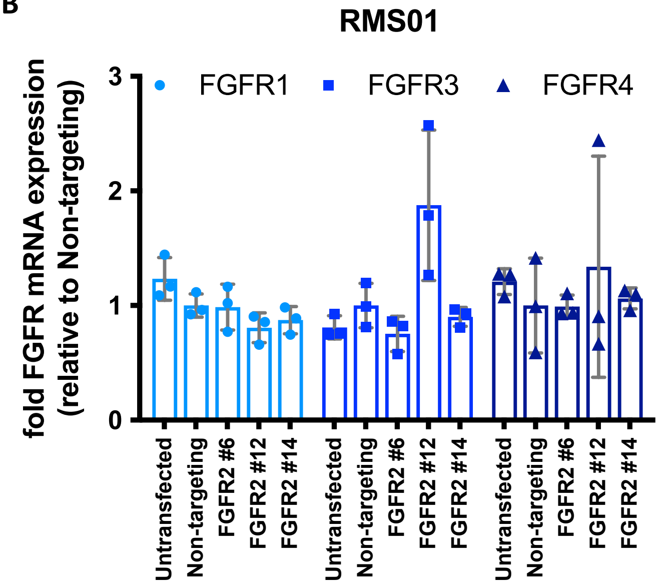

	FGFR1	FGFR3	FGFR4
NTC vs FGFR2 #6	n.s.	n.s.	n.s.
NTC vs FGFR2 #12	n.s.	*	n.s.
NTC vs FGFR2 #14	n.s.	n.s.	n.s.

D

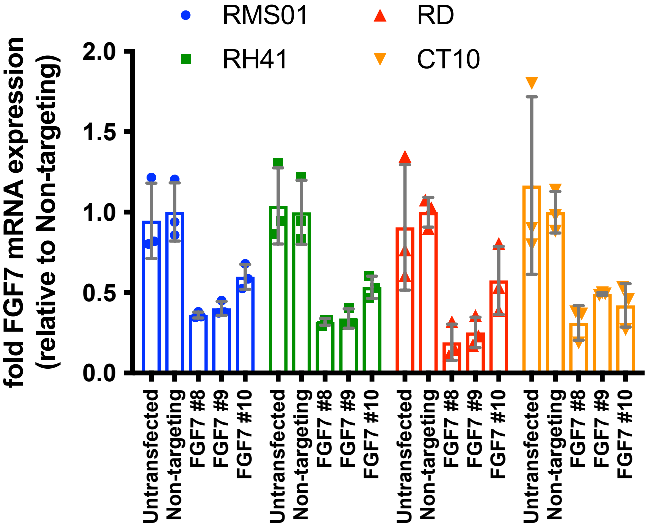

	RMS01	RH41	RD	CT10
NTC vs FGF7 #8	****	****	***	**
NTC vs FGF7 #9	***	****	**	*
NTC vs FGF7 #10	*	**	n.s.	*

Supplementary Figure 6. Validation of *FGFR2* and *FGF7* knockdown.

Reduction in *FGFR2* (**A**) or *FGFR1,3* and *4* (**B** and **C**) mRNA, as measured by qRT-PCR, after 72 hours of knockdown using three independent siRNAs to *FGFR2* in the indicated cell lines. (**D**) *FGF7* mRNA levels, as measured by qRT-PCR, after 72 hours *FGF7* knockdown by three independent siRNAs. in the indicated cell lines. Results are representative of three independent experiments with error bars representing standard deviation. Significance of differences were measured using One-Way ANOVA with Dunnetts multiple testing correction. NTC = Non-targeting control (n.s. = not significant, * = p<0.05, ** = p<0.01, *** = p<0.005, **** = p<0.001).

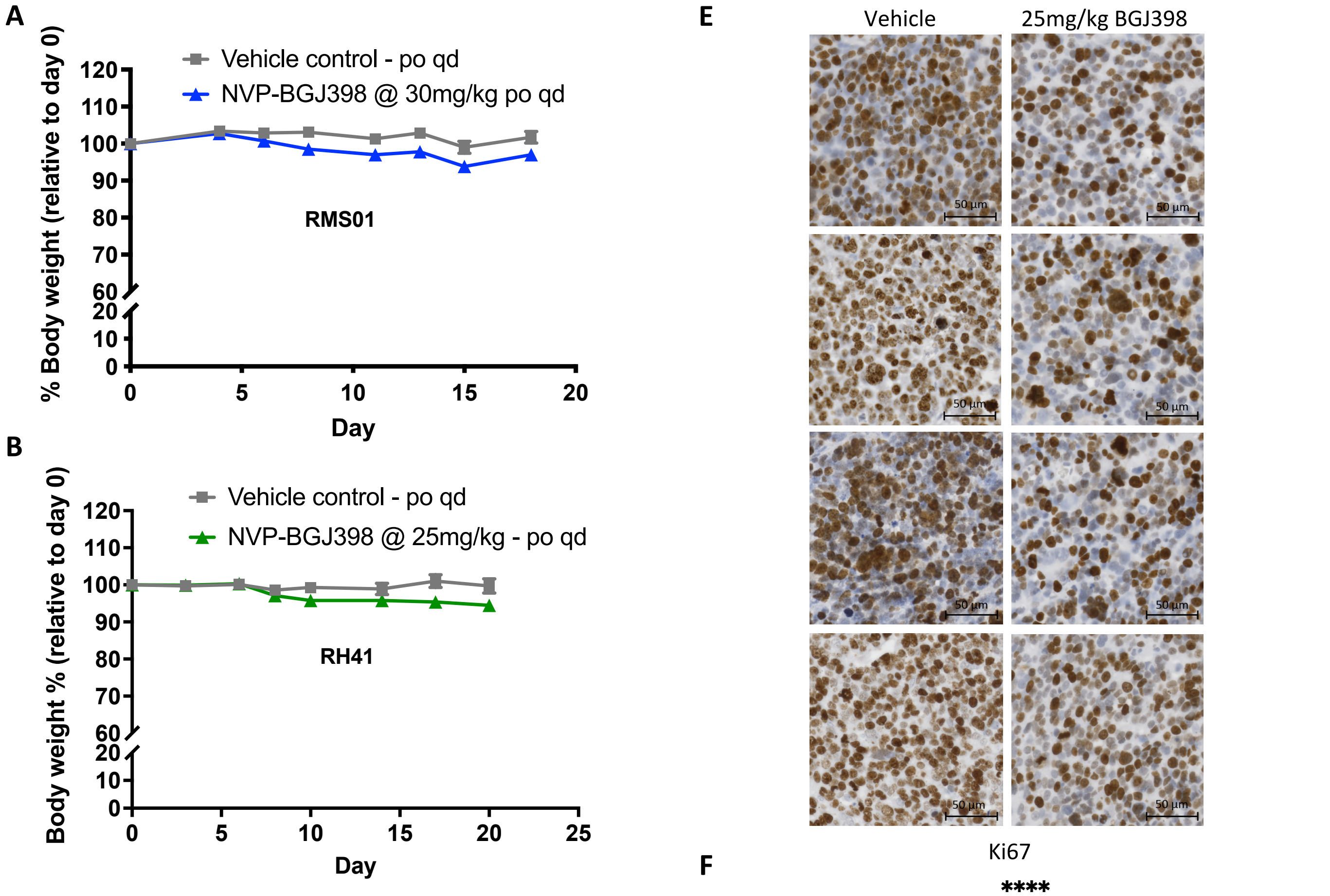

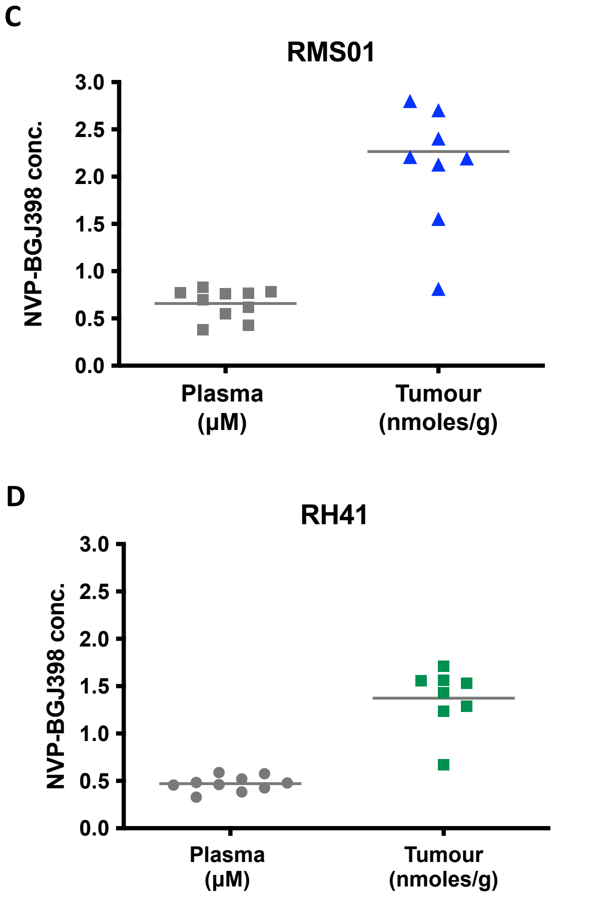

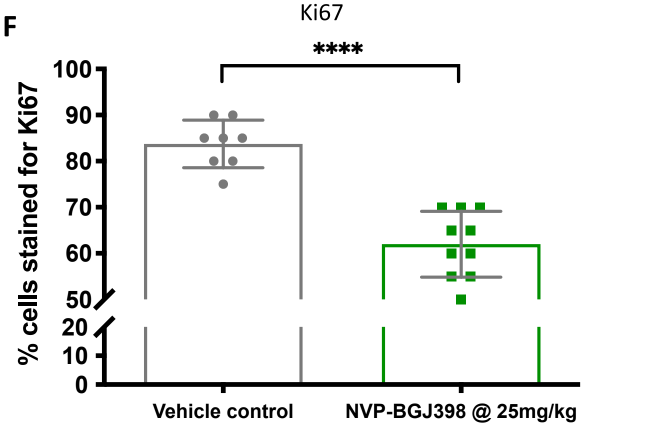

Supplementary Figure 7. NVP-BGJ398 *in vivo*

Mean body weight of NOD/SCID mice bearing RMS-01 (**A**) and RH41 (**B**) tumor xenografts after dosing with 30mg/kg or 25mg/kg NVP-BGJ398 q.d. for 15 or 20 days respectively. Accumulation of compound in RMS-01 (**C**) and RH41 (**D**) tumors compared to plasma after relevant dosing (see above). Error bars represent standard error of the mean. Control groups = 10 mice (RMS01) and 8 mice (RH41); treated groups = 10 mice (both RMS01 and RH41). (**E**) Representative images of Ki67 staining by IHC on sections from mice bearing RH41 xenografts and exposed to either vehicle or 25mg/kg BGJ398. Scale bar = 50µM n=8/group (**F**) Quantification of Ki67 positive cells from E. Significance of differences are measured using unpaired t-tests with Welch's correction. (**** = p<0.001)

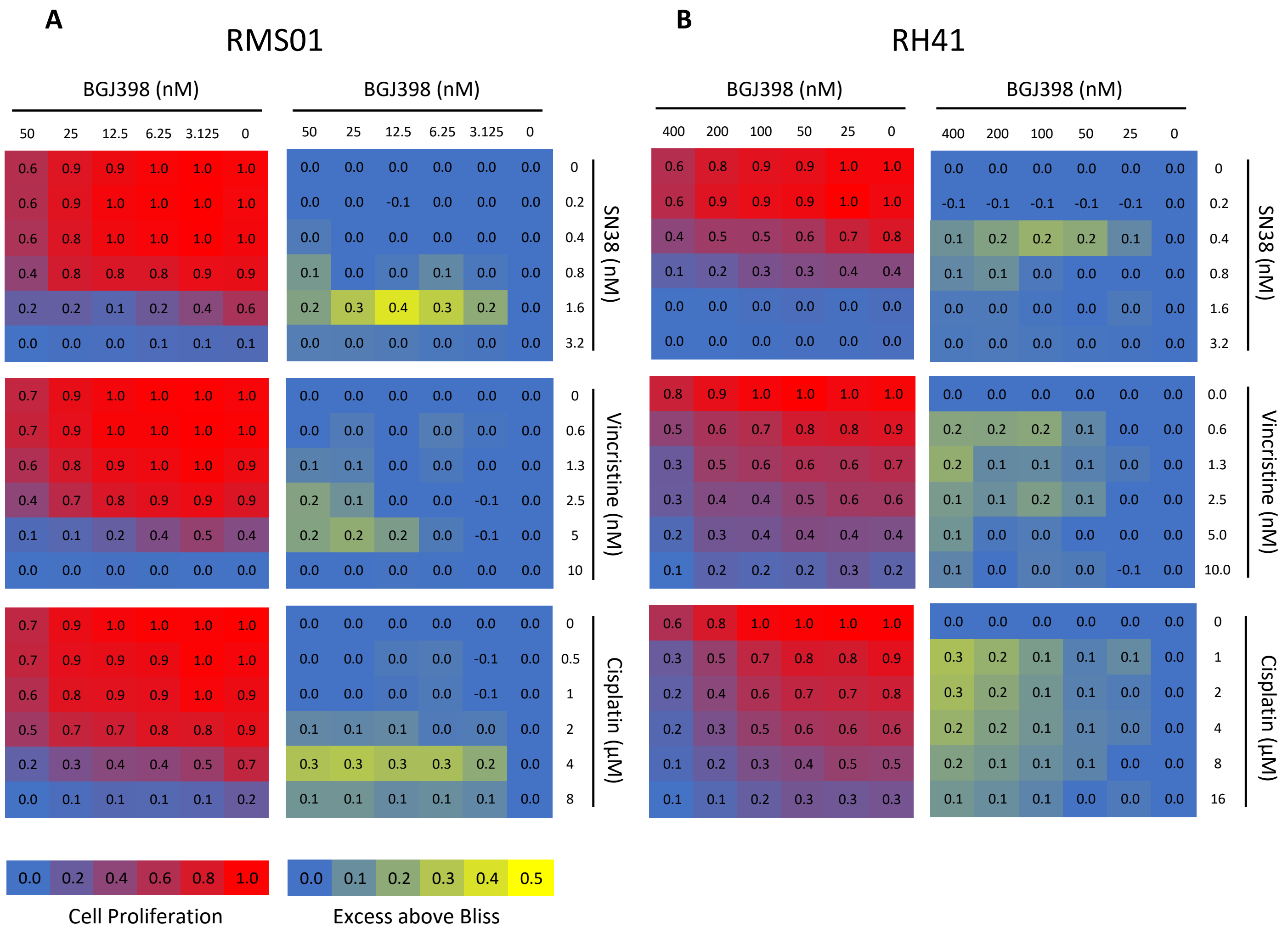

Supplementary Figure 8. NVP-BGJ398 synergises with SN38 and other DNA damaging agents

Heatmaps of RMS01 (**A**) and RH41 (**B**) cells exposed to a matrix of concentrations of NVP-BGJ398 and the indicated chemotherapeutics for 144 hours in 96 well plates. Proliferation in red and blue is normalized to DMSO treated controls with the excess above Bliss score in yellow and blue. Results are the mean from three independent experiments.

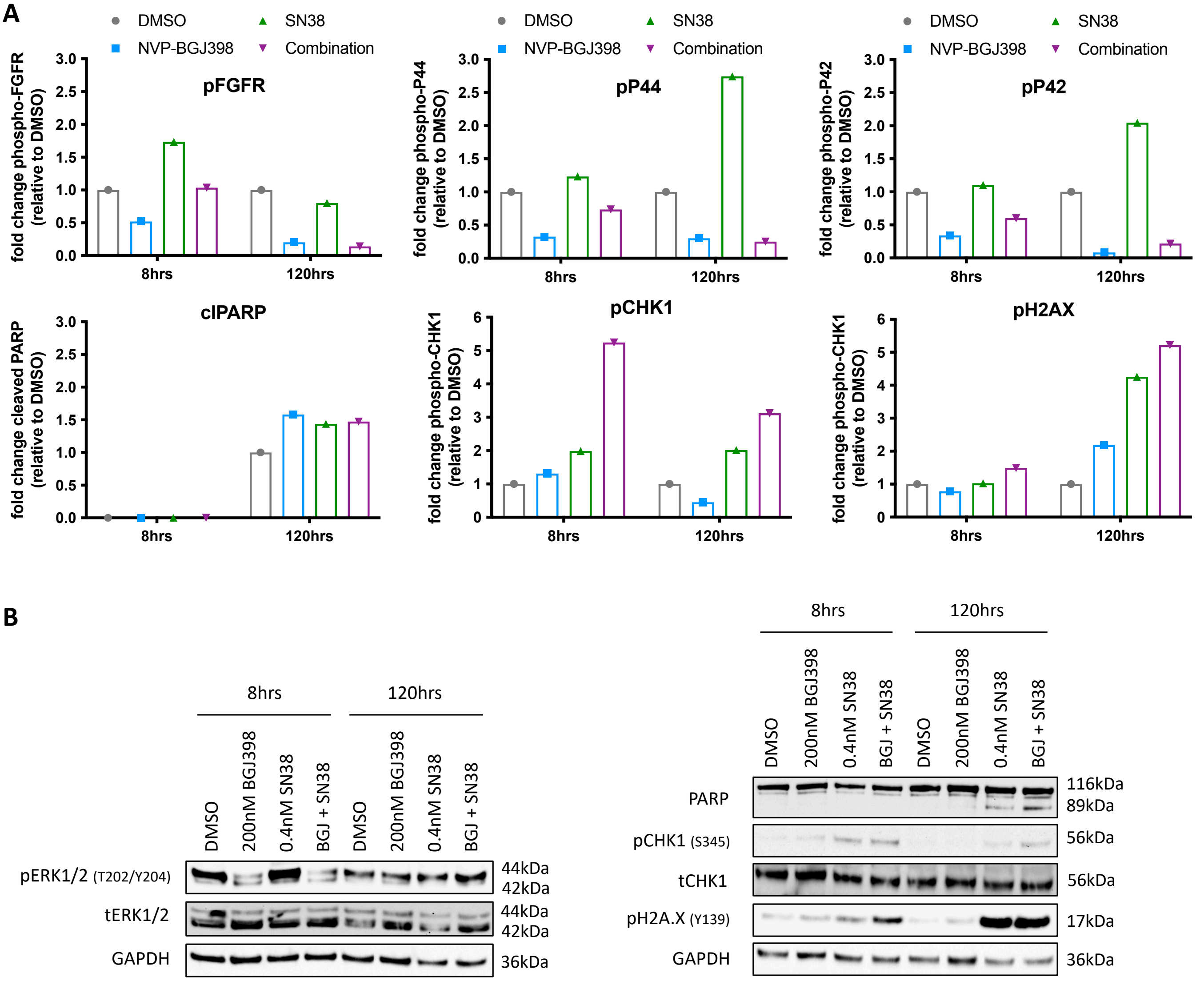

Supplementary Figure 9. Molecular markers of response to NVP-BGJ398, SN38 or the combination
(**A**) Quantification of the indicated proteins from RMS01 cells exposed to either 0.1% DMSO, 25nM NVP-BGJ398, 1nM SN38 or 25nM BGJ398 + 1nM SN38 (combination) for 8 hours or 120 hours (from blots in main figure 6D). Phospho proteins were taken as a ratio of the total, which was then normalised to GAPDH before being expressed as a fold change from DMSO. Mean of two independent experiments is shown. (**B**) Representative blots of signaling proteins in RH41 cells after 8 hours or 120 hours exposure to either NVP-BGJ398, SN38 or the combination of NVP-BGJ398 and SN38 as indicated. Results are representative of two independent experiments.
