# Supplementary material for: FGF7–FGFR2 autocrine signaling increases growth and chemoresistance of fusion‐positive rhabdomyosarcomas
Source: Mol Oncol. 2021 Dec 18;16(6):1272–89. doi: 10.1002/1878-0261.13145 (PMC8936514; doi:10.1002/1878-0261.13145)
Supplement: Supplementary file 2 — Table S1. List of antibodies used for immunoblotting. Table S2. Potency and selectivity profiles for FGFR inhibitors against a panel of protein targets. Table S3. RMS cell line response to NVP‐BGJ398 in 2D MTS assay. [file MOL2-16-1272-s002.pdf]

Supplementary Table 1.

Antibody	Phosphosite	Catalogue #	Vendor	RRID
Anti-phospho-tyrosine (p-Tyr-100)	generic	9411	Cell Signaling Technology	AB_331228
Anti-phospho-FGFR	Y653/654	3471	Cell Signaling Technology	AB_331072
Anti-phospho-ERK1/2	T202/Y204	9101	Cell Signaling Technology	AB_331646
Anti-phospho-STAT3	Y705	9145	Cell Signaling Technology	AB_2491009
Anti-phospho-AKT	S473	9271	Cell Signaling Technology	AB_329825
Anti-phospho-CHEK1	S345	2348	Cell Signaling Technology	AB_331212
Anti-phospho-H2A.X	S139	05-636-I	Merck Millipore	AB_2755003
Anti-ERK1/2	-	9102	Cell Signaling Technology	AB_330744
Anti-STAT3	-	9139	Cell Signaling Technology	AB_331757
Anti-AKT	-	9272	Cell Signaling Technology	AB_329827
Anti-PARP	-	9542	Cell Signaling Technology	AB_2160739
Anti-CHEK1	-	2360	Cell Signaling Technology	AB_2080320
Anti-FGFR2	-	sc-122	Santa Cruz Biotechnology	AB_631509

Anti-FGFR4	-	sc-124	Santa Cruz Biotechnology	AB_631512
Anti-Topoisomerase IIα (D10G9)	-	12286	Cell Signaling Technology	AB_2797871
Anti-GAPDH (6C5)	-	MAB374	Merck Millipore	AB_2107445

Supplementary Table 2. Potency and selectivity profiles for FGFR inhibitors against a panel of protein targets. Protein targets are listed in the left column of the table with their biochemical IC50 values (nM). Figures are sourced from References 61,34,62,63

	ponatinib	NVP-BGJ398	dovitinib	nintedanib
FGFR1	2.2	0.9	8	69
FGFR2	1.6	1.4	-	37
FGFR3	18.2	1	9	108
FGFR4	7.7	60	-	610
VEGFR1	3.7	-	10	34
VEGFR2	1.5	180	13	21
VEGFR3	2.3	-	8	13
FLT3	12.6	-	1	26
CSF-1R	-	-	36	-
PDGFRβ	7.7	-	27	65
PDGFRα	1.1	-	210	59
C-Kit	12.5	-	2	-

Supplementary Table 3. RMS cell line response to NVP-BGJ398. Cell line GI50 values as assessed by MTS assay after 72 hours exposure to NVP-BGJ398. Dose response curves with their associated R2 and GI50 values were evaluated using non-linear regression with a variable slope in Graph Pad Prism (v7).

Cell Line	Fusion status	GI_{50} (μM)	R^2 values
RMS01	FP-RMS	0.09	0.94
RH4	FP-RMS	0.21	0.99
SCMC	FP-RMS	0.45	0.96
RMSYM	FN-RMS	0.67	0.96
RH41	FP-RMS	0.77	0.88
RMS559	FN-RMS	2.35	0.93
RD	FN-RMS	2.57	0.99
JR1	FN-RMS	3.56	0.98
TE617.T	FN-RMS	4.31	0.92
RH30	FP-RMS	4.77	0.99
TE381.T	FN-RMS	5.50	0.94
CT10	FN-RMS	5.55	0.89
RUCH3	FN-RMS	16.49	0.92

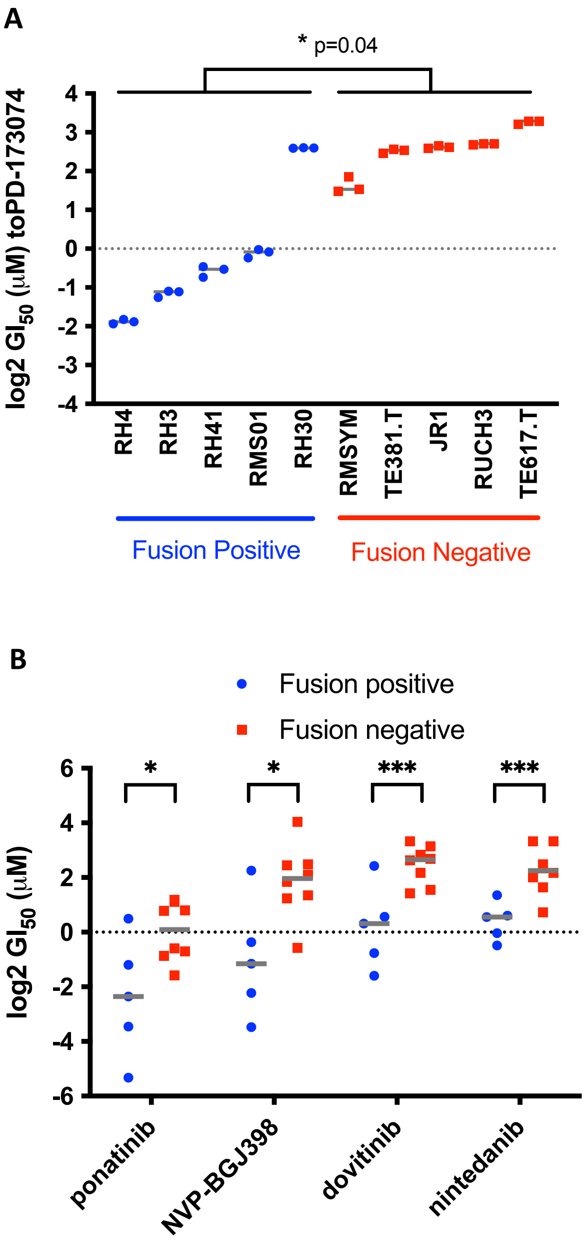

Supplementary Figure 1. 2D screening of RMS cells against FGFR inhibitors. (**A**) Waterfall plot of log2 GI_{50}s of PAX3-FOXO1 fusion positive (FP, blue) and fusion negative (FN, red) RMS cell lines to PD-173074 after 72 hours in 2D culture. (**B**) Dot plot of 2D cultured FP-RMS (blue) and FN-RMS (red) cell line log2 GI_{50}s for each of the compounds shown. Viability was measured after 72hrs by MTS. Results are representative of three independent experiments with error bars representing standard deviation. Significance of differences were assessed using unpaired t-tests with Welch's correction. *= $p<0.05$, ***= $p<0.005$.

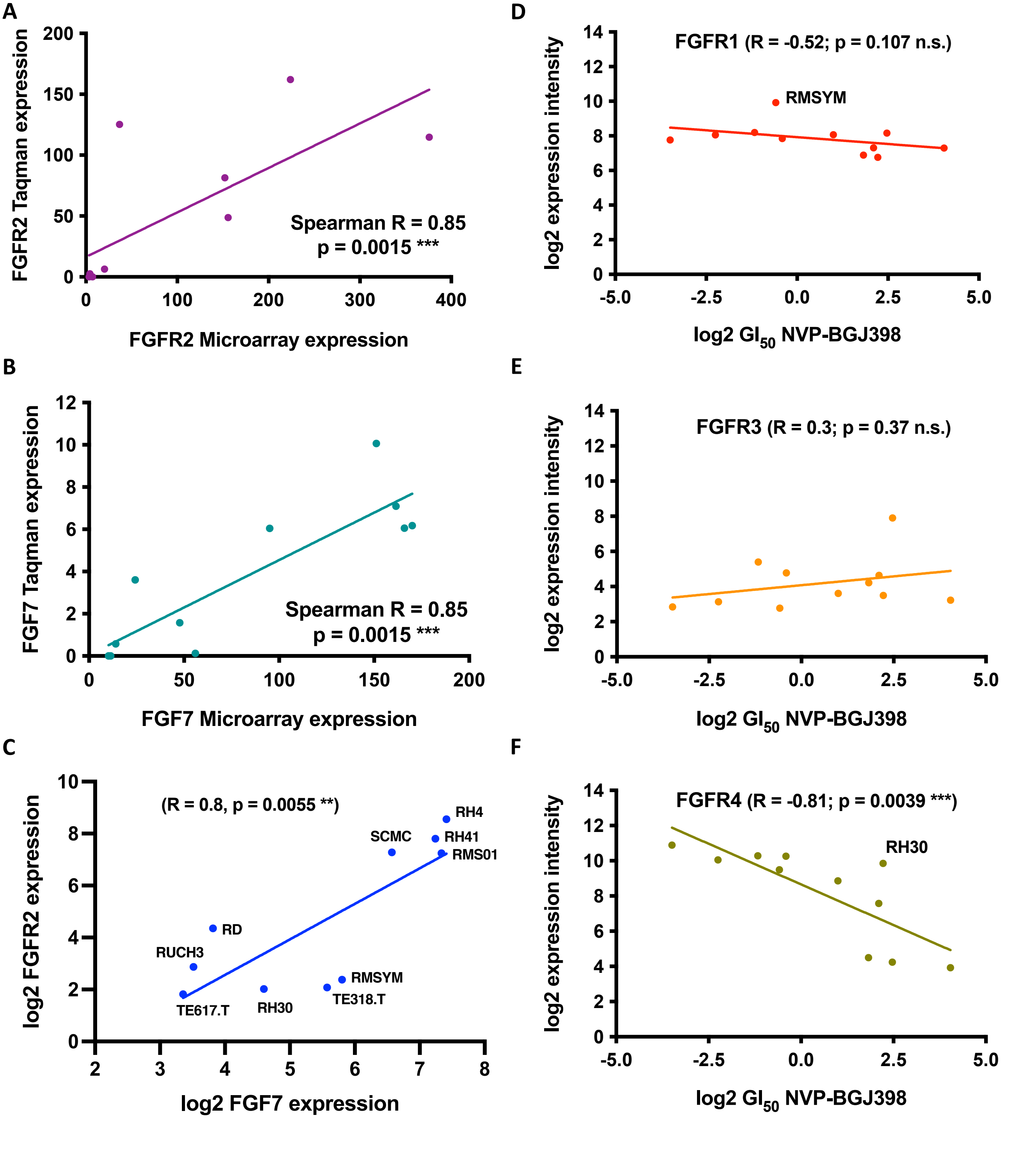

Supplementary Figure 2. Correlation between RMS cell line *FGFR* mRNA expression and log2 GI_{50} for NVP-BGJ398. Scatter plots showing the correlation between Affymetrix microarray and qRT-PCR (Taqman) measurements of *FGFR2* (**A**) (plum) and *FGF7* (**B**) (teal) mRNA in RMS cell lines. Taqman expression is fold change relative to normal skeletal muscle and microarray expression is gcrma normalised intensity. (**C**) Correlation between log2 *FGFR2* and *FGF7* mRNA expression as measured by Affymetrix microarray for RMS cell lines. Scatter plots of *FGFR1* (**D**), *FGFR3* (**E**) and *FGFR4* (**F**) expression as measured by Affymetrix microarray, with log2 GI_{50} for NVP-BGJ398. The line of best fit, spearman r and associated p values are indicated on each graph.

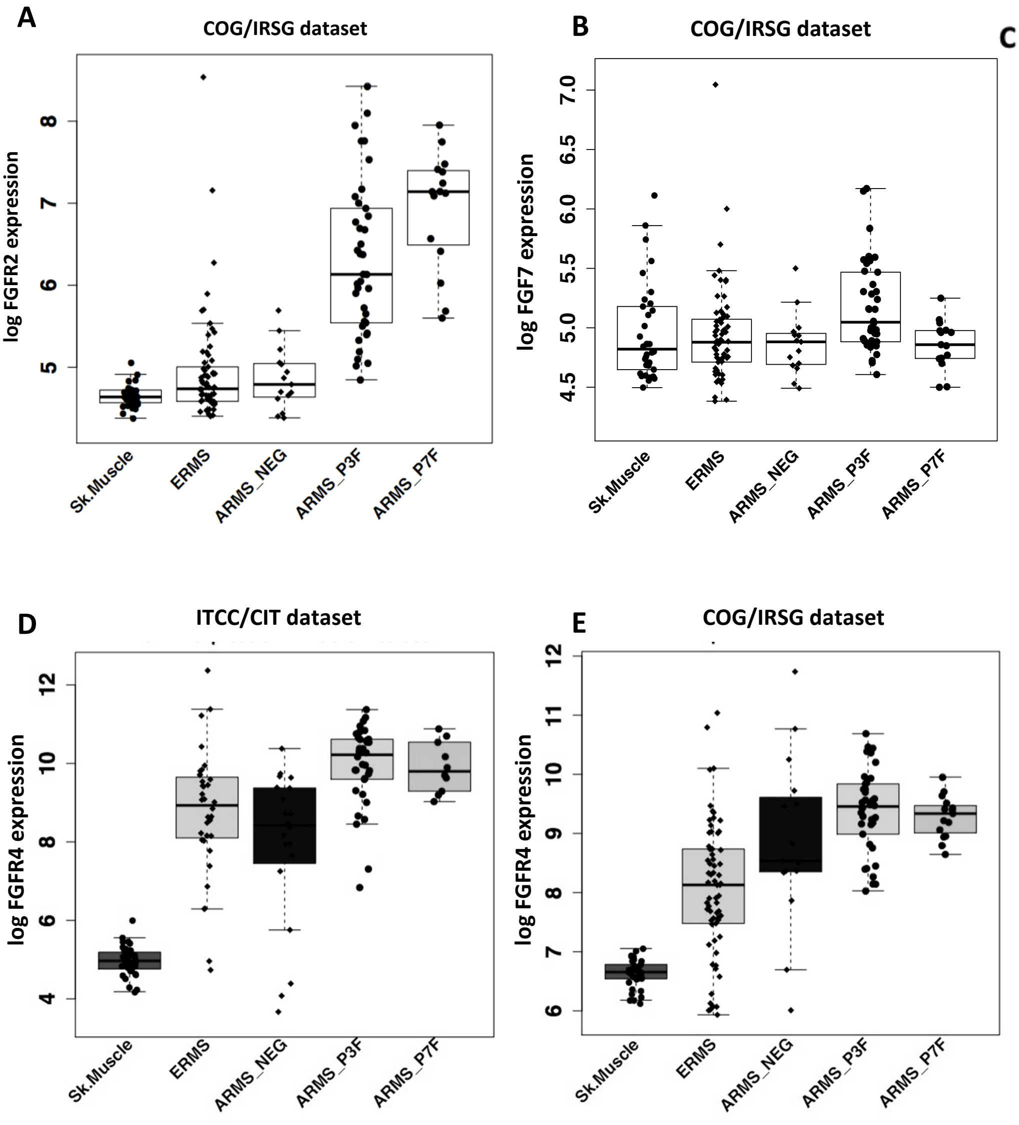

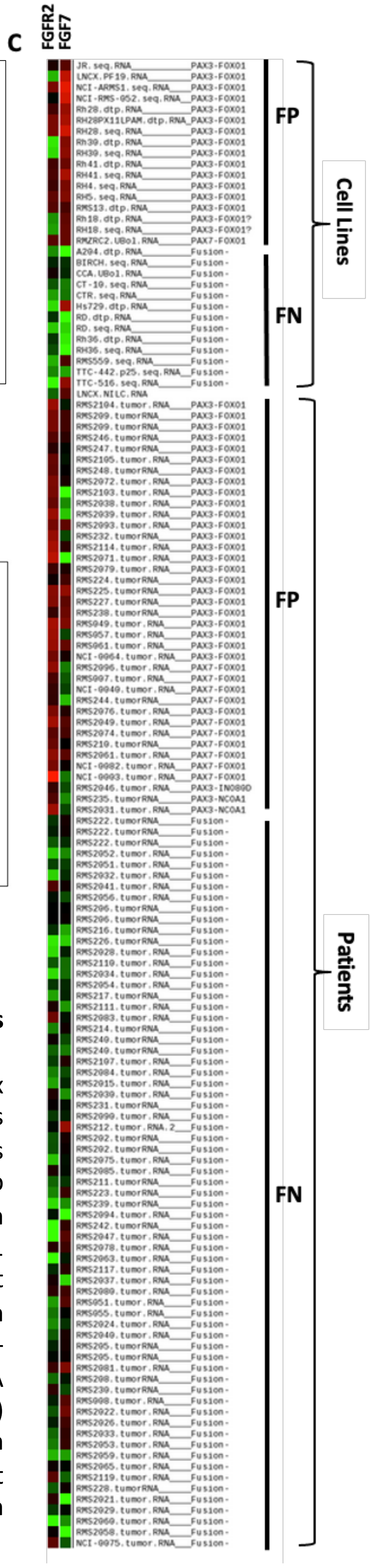

Supplementary Figure 3. ***FGFR2*, *FGF7 and FGFR4*** **mRNA expression is highly expressed in FP-RMS patient samples**

FGFR2 (**A**) and *FGF7* (**B**) mRNA expression, as measured by Affymetrix microarray, is significantly greater in FP-RMS (P3F; PAX3-FOXO1) patients compared to FN-RMS (ERMS and ARMS-NEG) from the Children's Oncology Group/Intergroup Rhabdomyosarcoma Study Group (COG/IRSG) cohort. (n=139 patients) Wilcoxon rank sum test for both genes is p<0.001. (**C**) RNASeq data (from Shern, J.F. *et al* 4(2):216-31 2014) validates findings from Affymetrix microarrays that FP-RMS patient samples and cell lines harbour greater *FGFR2* and *FGF7* expression compared to FN-RMS samples and cell lines. (n=29 FP-RMS and 51 FN-RMS patients). (**D** and **E**) *FGFR4* mRNA expression as measured by cDNA microarray from patient samples in the ITCC/CIT (**D**) and COG/IRSG (**E**) cohorts. All RMS samples have higher *FGFR4* expression compared with normal skeletal muscle (Wilcoxon rank sum test, P=<0.001) but is highest in fusion positive RMS (Wilcoxon rank sum test, P=<0.001) in both cohorts.

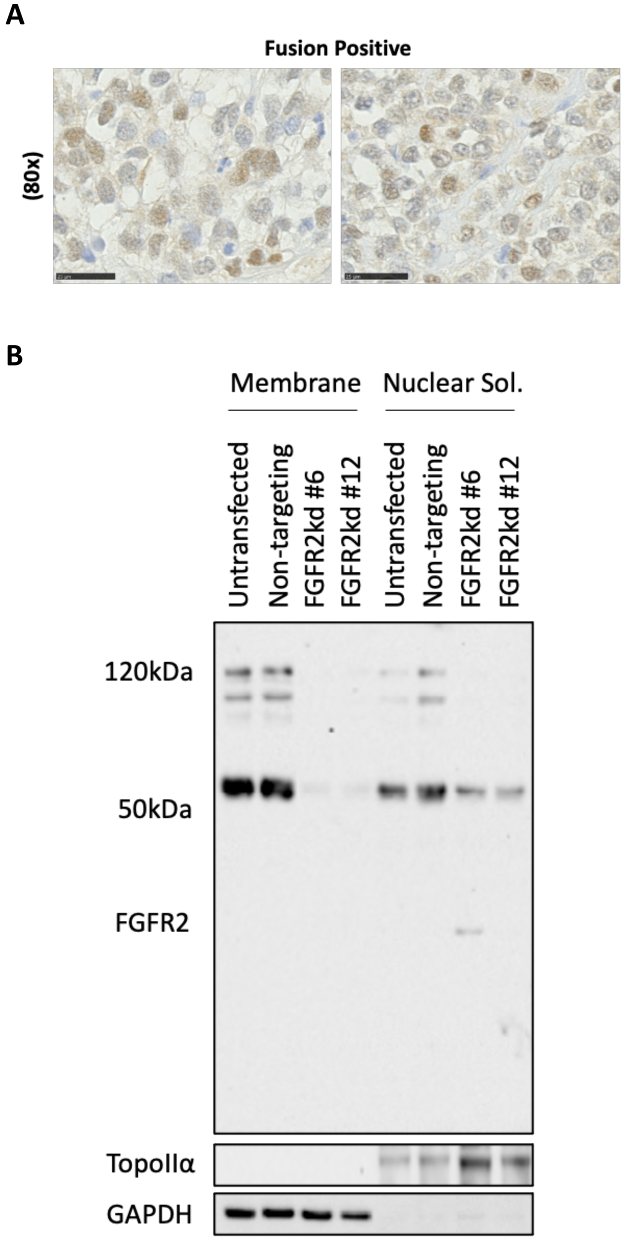

Supplementary Figure 4. FGFR2 is found in the nucleus of cells from FP-RMS patients and cell lines. (**A**) Representative images of nuclear FGFR2 staining in FP-RMS patients at 80x magnification. Scale bars represent 25µm. Image is representative of 12 FP-RMS samples. (**B**) Representative Western blot of FGFR2 in different subcellular fractions from RMS01 cells subjected to FGFR2 kd for 72 hours. TopoIIα and GAPDH were used as nuclear and membrane fraction specific markers respectively. Results are representative of two independent experiments.

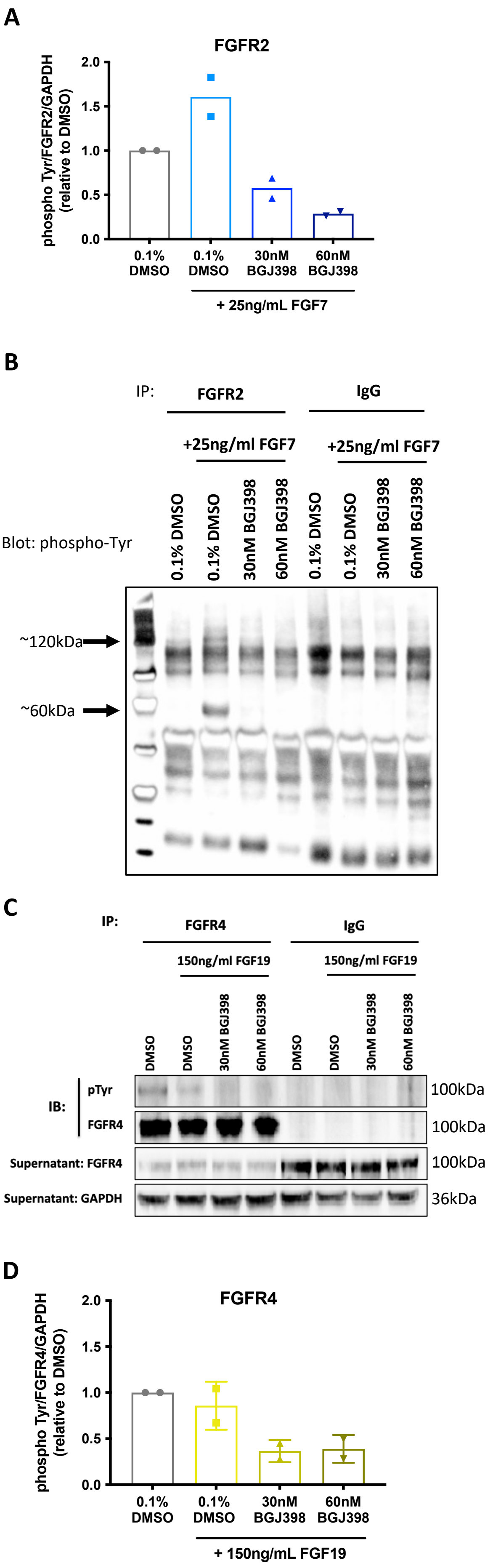

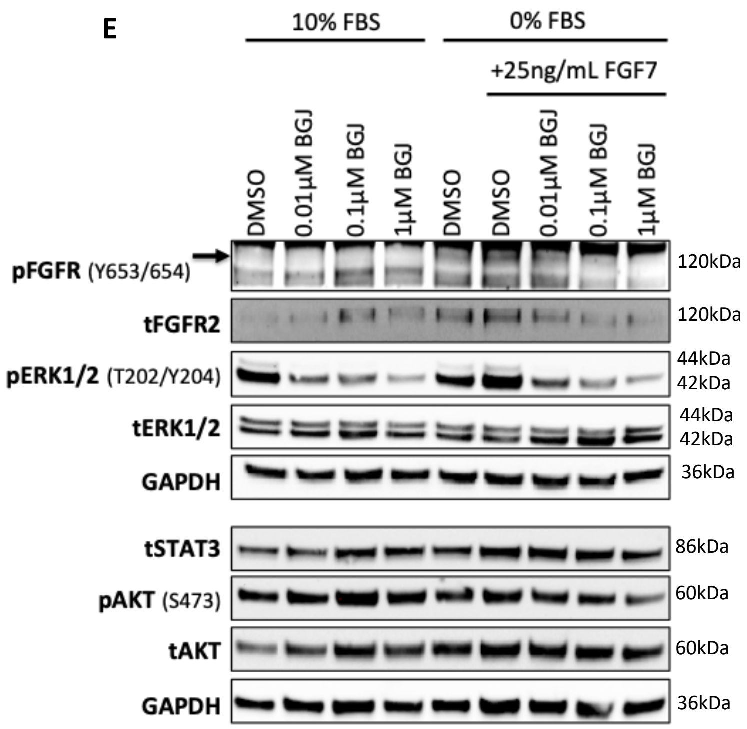

Supplementary Figure 5. Effects of FGF7 and NVP-BGJ398 on FGFR signalling in RMS cells.
(**A**) Quantitation of FGFR2 tyrosine phosphorylation in RMS01 cells exposed to FGF7 and NVP-BGJ398 (from western blot in Figure 3A). The ratio of tyrosine phosphorylation to total FGFR2 pulled down was normalised to the ratio of total FGFR2 pulled down to GAPDH. Resulting ratios for each treatment were then normalised to unstimulated cells with DMSO alone. (**B**) Full blot of tyrosine phosphorylation on FGFR2 immunoprecipitated from RMS01 cells exposed to DMSO or NVP-BGJ398 for 3 hours and 25ng/mL FGF7 for the final 20 minutes. Bands indicate long (~120kDa) and short (~60kDa) forms of FGFR2 (**C**) IP of FGFR4 from RMS01 cells exposed to DMSO or NVP-BGJ398 for 3 hours and 150ng/mL FGF19 for the final 20 minutes. (**D**) Quantification of blot B. Normalisation was conducted as described in A for FGFR2. (**E**) RH41 cells were cultured in 10% FBS or starved (0% FBS) for 16 hours before exposure to a range of concentrations of NVP-BGJ398 for 3 hours. Cells in 0% FBS were exposed to 25ng/mL FGF7 for the last 20 minutes of drug treatment before being lysed, and the phosphorylation status of several signalling proteins assessed by western blot. The band corresponding to phosphorylation of FGFR is indicated by the arrow in the uppermost panel. N=2

A

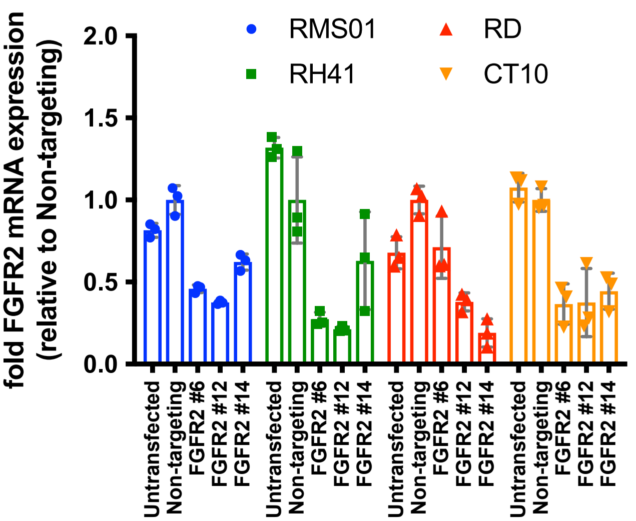

	RMS01	RH41	RD	CT10
NTC vs FGFR2 #6	****	***	*	****
NTC vs FGFR2 #12	****	***	****	****
NTC vs FGFR2 #14	****	n.s.	****	***

C

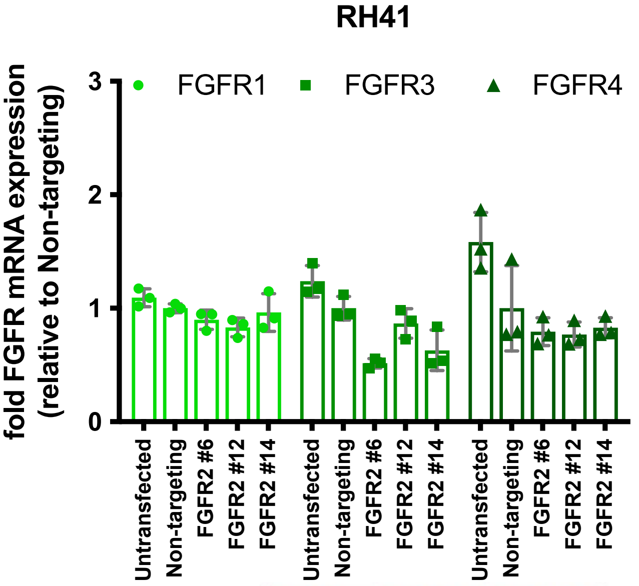

	FGFR1	FGFR3	FGFR4
NTC vs FGFR2 #6	n.s.	***	n.s.
NTC vs FGFR2 #12	n.s.	n.s.	n.s.
NTC vs FGFR2 #14	n.s.	*	n.s.

B

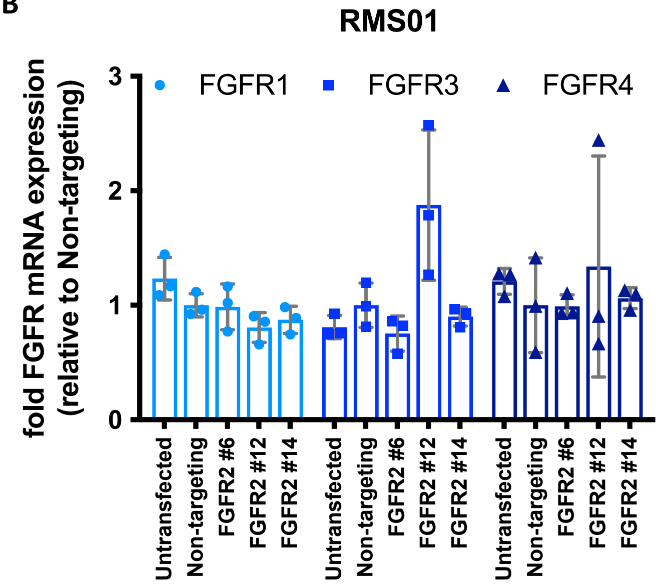

	FGFR1	FGFR3	FGFR4
NTC vs FGFR2 #6	n.s.	n.s.	n.s.
NTC vs FGFR2 #12	n.s.	*	n.s.
NTC vs FGFR2 #14	n.s.	n.s.	n.s.

D

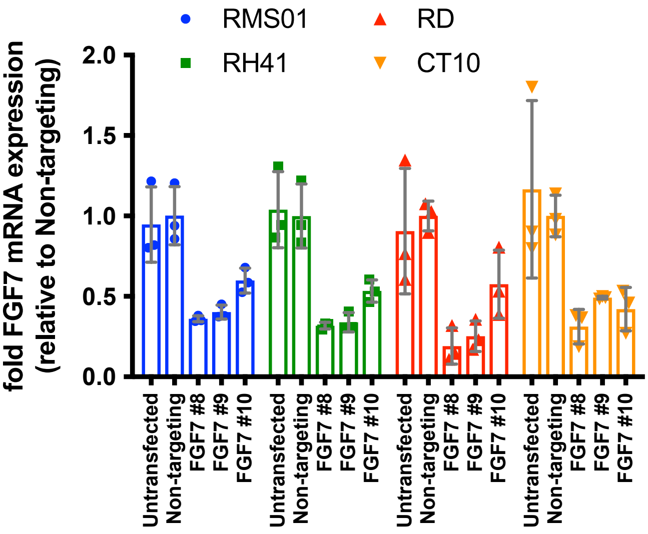

	RMS01	RH41	RD	CT10
NTC vs FGF7 #8	****	****	***	**
NTC vs FGF7 #9	***	****	**	*
NTC vs FGF7 #10	*	**	n.s.	*

Supplementary Figure 6. Validation of *FGFR2* and *FGF7* knockdown.

Reduction in *FGFR2* (**A**) or *FGFR1,3* and *4* (**B** and **C**) mRNA, as measured by qRT-PCR, after 72 hours of knockdown using three independent siRNAs to *FGFR2* in the indicated cell lines. (**D**) *FGF7* mRNA levels, as measured by qRT-PCR, after 72 hours *FGF7* knockdown by three independent siRNAs. in the indicated cell lines. Results are representative of three independent experiments with error bars representing standard deviation. Significance of differences were measured using One-Way ANOVA with Dunnetts multiple testing correction. NTC = Non-targeting control (n.s. = not significant, * = $p<0.05$, ** = $p<0.01$, *** = $p<0.005$, **** = $p<0.001$).

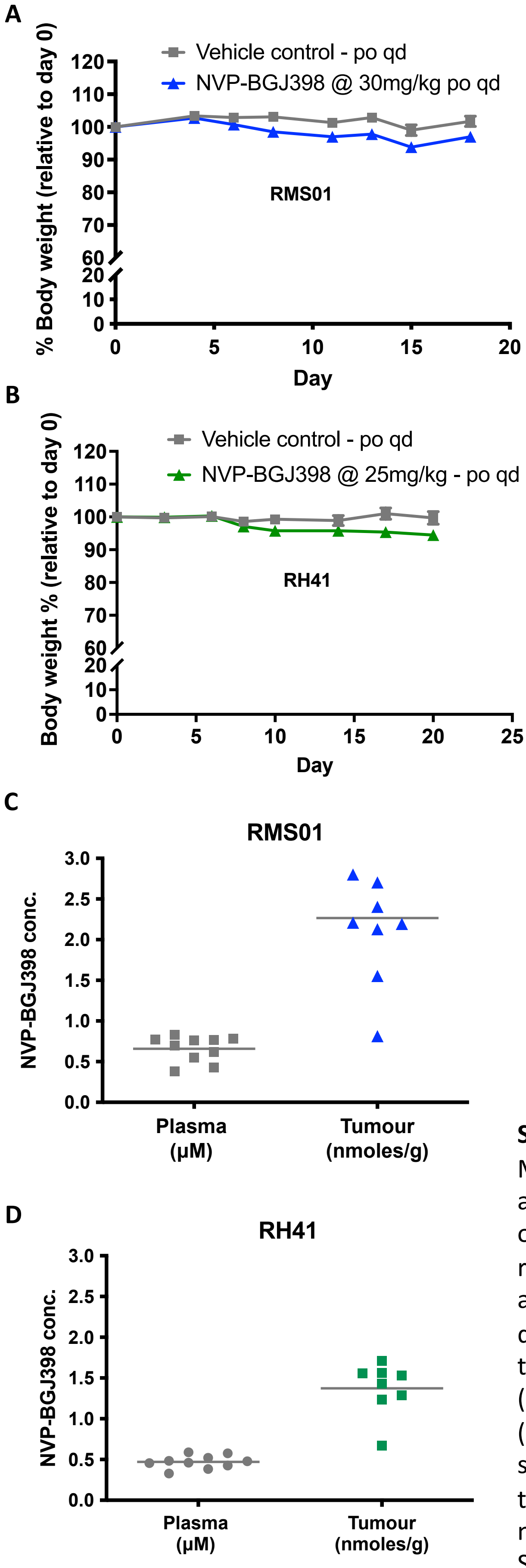

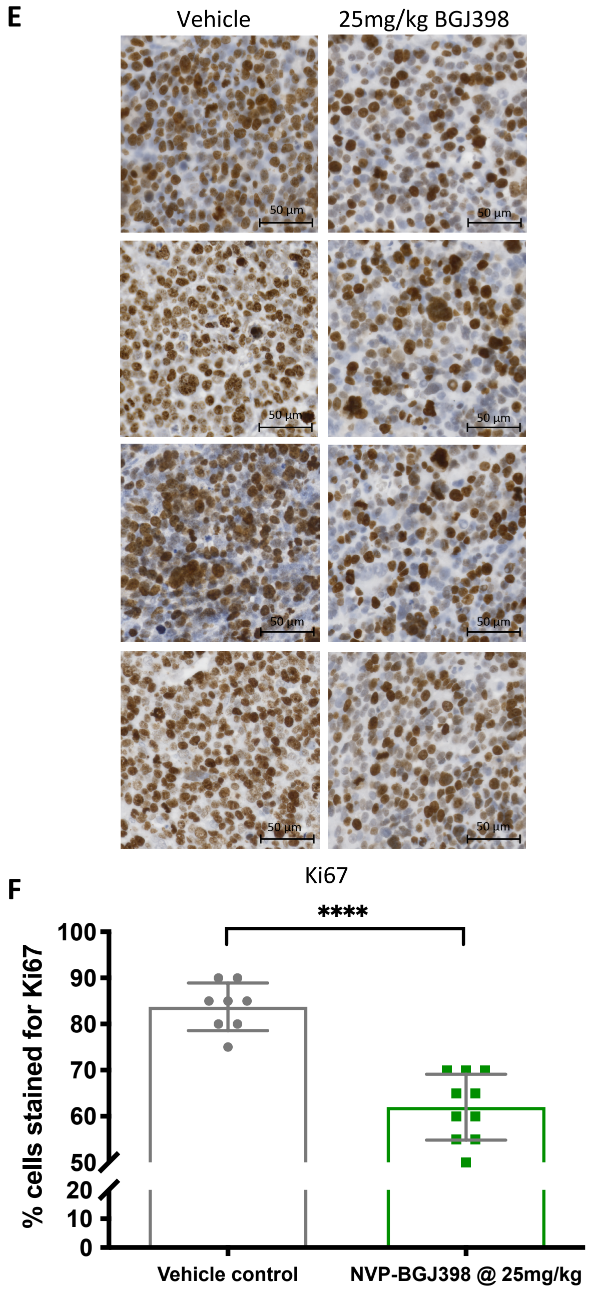

Supplementary Figure 7. NVP-BGJ398 *in vivo*
Mean body weight of NOD/SCID mice bearing RMS-01 (**A**) and RH41 (**B**) tumor xenografts after dosing with 30mg/kg or 25mg/kg NVP-BGJ398 q.d. for 15 or 20 days respectively. Accumulation of compound in RMS-01 (**C**) and RH41 (**D**) tumors compared to plasma after relevant dosing (see above). Error bars represent standard error of the mean. Control groups = 10 mice (RMS01) and 8 mice (RH41); treated groups = 10 mice (both RMS01 and RH41). (**E**) Representative images of Ki67 staining by IHC on sections from mice bearing RH41 xenografts and exposed to either vehicle or 25mg/kg BGJ398. Scale bar = 50µM n=8/group (**F**) Quantification of Ki67 positive cells from E. Significance of differences are measured using unpaired t-tests with Welch's correction. (**** = p<0.001)

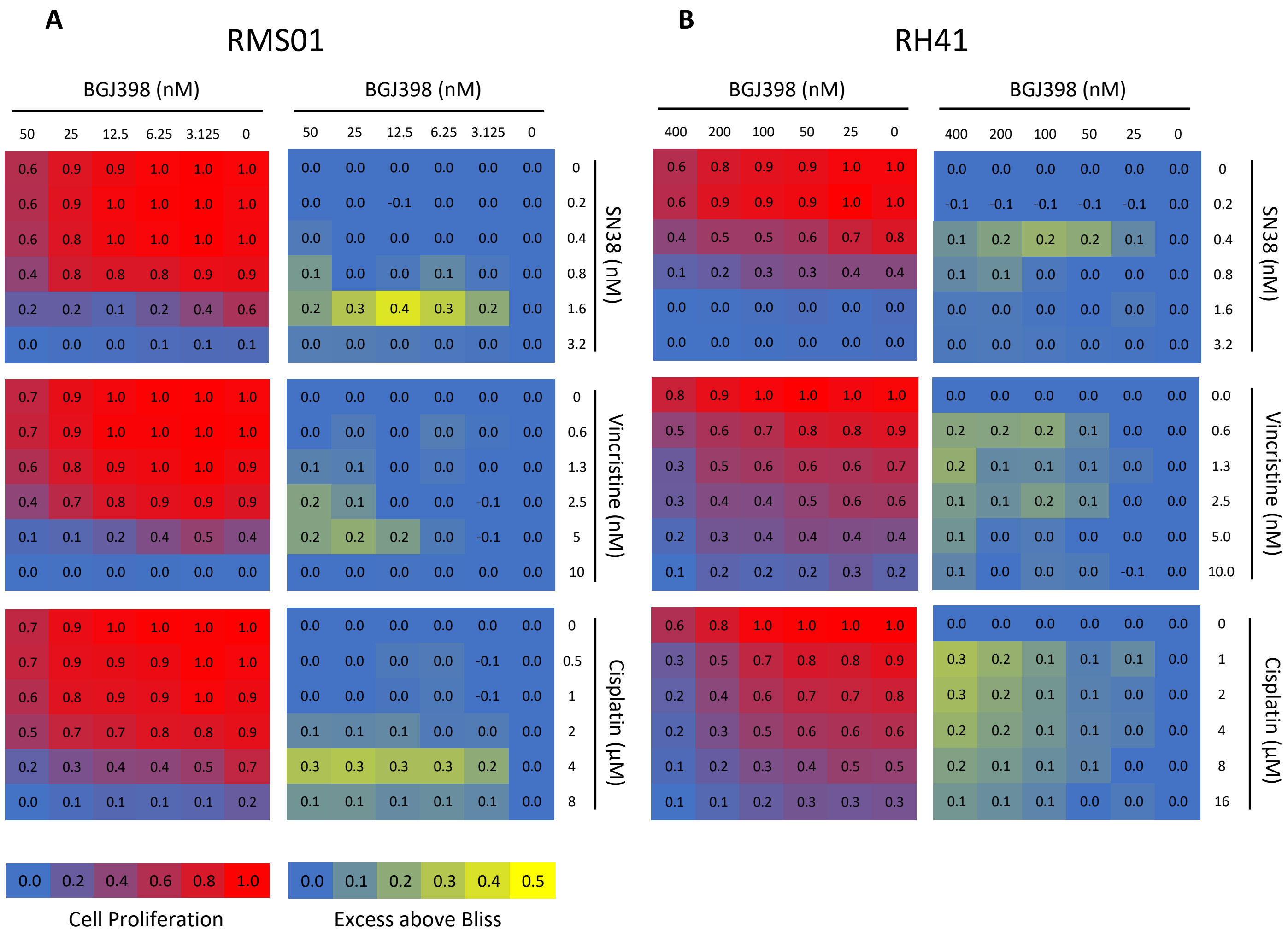

Supplementary Figure 8. NVP-BGJ398 synergises with SN38 and other DNA damaging agents
Heatmaps of RMS01 (**A**) and RH41 (**B**) cells exposed to a matrix of concentrations of NVP-BGJ398 and the indicated chemotherapeutics for 144 hours in 96 well plates. Proliferation in red and blue is normalized to DMSO treated controls with the excess above Bliss score in yellow and blue. Results are the mean from three independent experiments.

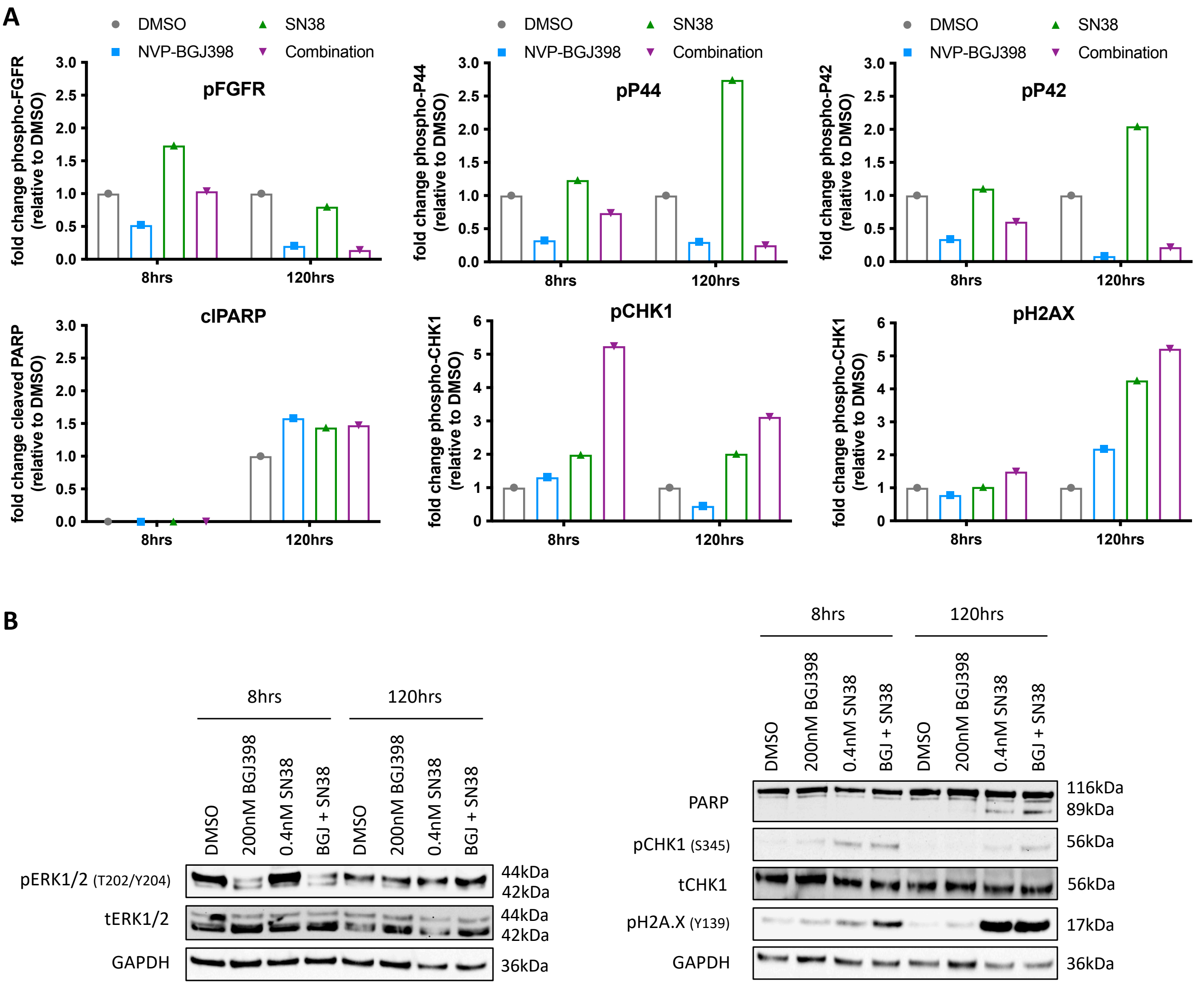

Supplementary Figure 9. Molecular markers of response to NVP-BGJ398, SN38 or the combination
(**A**) Quantification of the indicated proteins from RMS01 cells exposed to either 0.1% DMSO, 25nM NVP-BGJ398, 1nM SN38 or 25nM BGJ398 + 1nM SN38 (combination) for 8 hours or 120 hours (from blots in main figure 6D). Phospho proteins were taken as a ratio of the total, which was then normalised to GAPDH before being expressed as a fold change from DMSO. Mean of two independent experiments is shown. (**B**) Representative blots of signaling proteins in RH41 cells after 8 hours or 120 hours exposure to either NVP-BGJ398, SN38 or the combination of NVP-BGJ398 and SN38 as indicated. Results are representative of two independent experiments.
